# Supplementary material for: “If it is not made easy for me, I will just not bother”. A qualitative exploration of the barriers and facilitators to recycling plastics
Source: PLoS One. 2022 May 3;17(5):e0267284. doi: 10.1371/journal.pone.0267284 (PMC9064103; doi:10.1371/journal.pone.0267284)
Supplement: S1 File — (DOCX) [file pone.0267284.s001.docx]

**Supporting Information**

**S1 Interview Schedule**

The interview schedule will encourage reflections on current practice around purchasing and consuming of plastics and attitudes towards bio-based and recycled plastics. A small number of scenarios may be used to prompt **motivation, capabilit**y (knowledgeable, being in control), and **opportunity.**

(If a Focus Group is held, we would encourage participants to discuss the questions as a group).

This is the full set of questions.

| Questions | Prompts |
| --- | --- |
| (1)Please tell us a little bit about yourself, any family, do you normally do the shopping or help out with the shopping?  *(Encourage reflections on daily living and the role of plastic products).* | **C and O**  *Do you have a busy life - do you work for example?*  *Do you shop a lot, do you do all the shopping?*  *Do you keep to a budget – is that important?*  *(Encourage reflections on daily living and the role of plastic products).* |
| (2)What comes into your mind when you think of plastic and what we use it for? | **C and O**  *If they can’t think of anything – suggest thinking of food packaging, containers, children’s toys, building materials, window frames etc.* |
| (3) Who should do something about the problem of the build-up of plastic waste? | ***O M***  *Ask who do you think is responsible, do they feel any personal responsibility?*  *What about businesses, pubic, government etc*. |
| (4) Can you describe your plastic recycling activities? | ***C O M B***  *Ease with which they can separate waste, frequency, any barriers.(self efficacy, confidence)*  *local re-cycling service*  *Would incentives encourage you to recycle more, should government do more?* |
| (5) What is the relevance of recycling in your life - is it a priority for you at all, and, If not why not? | **M**  *What would it mean to you if you were unable to recycle for example?*  *Identity, values, moral responsibility* |
| (6) I have *here an empty clean plastic food container (*give to participant), would you separate this from the other waste try to the recycle this and what would you do with the different parts of it? | ***CO B***  *Test knowledge* ***behavioural skills*** *and understandings about different parts (lids, anything that can be peeled off etc.)*  *How easy is this to do*  *and how should/could it be improved so that you are more likely to recycle it?* |
| Questions | Prompts |
| (7) Do you ever actively seek information that may help you recycle more? | ***C M B***  *Have they looked for information on internet, phoned the council, any charity websites?* |
| (8) How do you feel when you report about plastic bottles washing up on beaches and plastic waste floating in the oceans? | **M**  *Awareness of media campaigns and the effects. David Attenborough documentaries etc.* |
| *Prompt for follow on questions –*  Currently most of the plastic we use is made from oil or petroleum. But the plastic or polymer industry is now pushing to develop alternatives to oil such as agricultural crops etc. They call these ‘bio-based plastics. |  |
| (9) What are your thoughts about replacing plastics made from petroleum with products manufactured using materials that are grown naturally such as corn for example? | *What pros or cons do you see with this new approach?*  *Do you think we can save resources using these materials?* |
| (10) Some agricultural materials such as blood, feathers, carcass, and litter actually contain the building blocks for new materials.  How would you feel about using these materials to package food?  How would you feel about using these materials for other things like carrying building materials or clothing? | **M** |
| (11) As new materials emerge onto the market they are often, initially, more expensive. Would you be willing to pay for a product made from bio-based polymers or plastics? | **C O M**  *How much if at all – 5% 10% 15% or more?* |
| (12) We would like to show you some demonstrator products that we prepared at QUB, can we ask you what you think about them?  Will you like to buy food wrapped with this kind of packaging?  (Yes/No)  Can you suggest changes in design? | **C O M B** |

| Questions | Prompts |
| --- | --- |
| (13) Can you envisage any issues with recycling these products? | **C O M B** |
| (14) What are your thoughts about the idea that the introduction of bio-plastics will solve the issues related to plastic waste and the pollution it causes? | **COM**  *Do you know what bio plastics are?*  *What resources do you think might help you and others to do more?*  *(Time, money, education, community initiatives etc.?* |
| (15) Would you buy, and use, food packaging made from sustainable plastics? | **COM**  *Do you know what bio plastics are?*  *What resources do you think might help you and others to do more?*  *(Time, money, education, community initiatives etc.?* |
| (16) Anything you would like to add that we haven’t covered? | *If they ask for more information – provide a sheet with internet sites where they can get more information.* |
