# Supplementary material for: “If it is not made easy for me, I will just not bother”. A qualitative exploration of the barriers and facilitators to recycling plastics
Source: PLoS One. 2022 May 3;17(5):e0267284. doi: 10.1371/journal.pone.0267284 (PMC9064103; doi:10.1371/journal.pone.0267284)
Supplement: S2 File — (DOCX) [file pone.0267284.s002.docx]

**Supporting Information**

**S2 Additional quotes to support each high level theme**

| **Capability** |
| --- |
| Confusion about what plastic can be recycled  *“I would be quite confident that I can recycle a yogurt pot or a tin or like a can from like a tin of beans and that type of thing or like beer bottles, the things that get me are when like you’ve got combinations of materials, like something with a film lid, like a plastic container with a film lid and like if you’ve already taken the outer packaging off something and you have thrown that away and you have kept the rest of it and you don’t have the little film can’t be recycled but the carton can, so yeah, those things I think in general are really the only ones which cause me any confusion”. ID3.*    “*I would say and obviously there is so much plastic that food comes in, that you're not sure, and what I would, what I tend to do, is to put it all in the blue recycling bin and kind of think oh well, if it can't be recycled then it’s somebody else's problem, which is maybe not the best attitude to have” ID1*  *“Yeah, so soft plastics I would be confused, about whether you can or can't cycle, and my take was that you can’t and things like your punnets for fruit, and yoghurt pots, I would be unsure about, and they would go in the black bin” ID8.*  *“I am never too sure whether bleach bottles or cleaning products go into it. But I put them in and I hope that I haven't contaminated”. ID18.*  *“There is also a confusing amount of information, so people are putting recyclable on packaging, because, for example it says on most nappies, recyclable, because theoretically they are recyclable, but in practice they cannot be recycled in this country”. ID11*  *“I recycle a lot, but I don't feel completely confident that I know exactly what can, and can’t be recycled. But mostly just when it comes to plastic really, I wouldn’t feel that way about tins or cardboard, or anything like that, it's the plastic that, you know, because there are so many different types of plastic that there are big question marks over it”. ID8*  *“I think there are so many different types of plastic as well on the market that, for the average householder, and even our recycling bins at the university, it is a bit bewildering as to what you can actually put into the plastic recycling, and what you can't, so there's a real lack of clarity around that, which then has challenges downstream for recycling because if you put lots of different types of plastic in, then you're actually causing more problems for the recyclers, in getting a good quality plastic material at the other end”. ID21*  *“It would be nice if everything was standardised so that it was recyclable or not, so you knew for sure, and you weren’t going over the bins and thinking, I don’t know which bin to put this in!”. ID20*  *“I think sometimes it is confusion with a lot of people, I know my husband it's taken me a long time to train my husband (laughing), about what is recyclable and what isn't”. ID17*  *“I think they (Local authority) are trying to do good things, but I think the website needs to be updated I think the website is confusing and I don't think that the information is quickly available”..ID24* |

| **Capability – continued)** |
| --- |
| There was little evidence of information-seeking behaviour, to reduce uncertainty  *“I am still doing what I can for example this (bag containing a pastry), I would have no idea if this was for the recycling, but I would throw it in, because it’s got paper on it, and this looks like plastic to me”. ID25*  *“When tired, I would never impose upon myself to go out of my way to find out more information about recycling, or go to more effort when I have individual resources depleted like I am tired, or things are not going well for me, if it is not made easy for me, I will just not bother”. ID23*  *“I think the limit of seeking out information is like reading the side of the packaging. Because, like, it’s that thing where normally if you are say, cooking, and you're throwing something in the bin, it’s second nature”. ID3*  *“So I think the main problem is plastic, I would say and obviously there is so much plastic that food comes in, that you're not sure, and what I tend to do, is to put it all in the blue recycling bin and kind of think oh well, if it can't be recycled then it’s somebody else's problem”. ID1*  *“So would you go on the council website, to look at recycling information (interviewer)?” “Not really no, I just went there so I could order my bin”. ID25*  *“I would buy them in loose plastic other times I would buy them, depending on the type of tomato it is, it may come with an under tray as well as the additional plastic on top of it. Now the under trays which tend to be a bit more rigid plastic, I’m not sure if they are 100% recyclable or not but I work under the assumption that they are, and it goes in my recycling bin.”ID10*  *“I don’t know why I think that it is better to put something that's recyclable into the main waste than to put something that's not recyclable into the recycling bin. I don't know where the attitude has come from, but I haven’t, it almost like a time issue, when you are throwing something in the bin that is just such a passive kind of action isn’t it, and then you don't really want to get out your phone be like, “can I recycle that?” (laughing), even though it's so easy nowadays to do that”. ID3*  *“I am still slightly confused to what is recyclable and yes, you can look on, you know look on bottles and stuff for some kind of logo or something, but it’s still, in some cases it’s still quite unclear, so recycle what we can”. ID12*  *“I don’t know if any of this is going to be recycled in (city), because if you are putting it in the recycling bin and the whole thing ends up being contaminated and being put to landfill, then we are not doing a good job. But now I have a big pile of black plastic in my house I don't know what to do with”. ID24*  *“Err, I think people really should be proactive because it really doesn't take that much effort and people are quite apathetic when it comes to these issues because they think, awe, why should I have to do it, err, you know. Or maybe there is a bit of a culture of, it really should be given to me, rather than seek it out and so I think, yeah people should, because it is such an important issue, people should really move it up a bit higher on the list of priorities”. ID1*  *“The percentage of people who were completely resistant was very small, … I think they're just set stone that they don't think it's their responsibility or they just plainly don't care”. ID1* |

| **Capability – continued)** |
| --- |
| Knowledge of environmental impact of plastic waste is limited to the harming of sea-life  *“When I'm thinking about plastic, the first thing I normally think about is all these stories about how animals are being harmed by the production of and wasting of plastic or I should say the incorrect disposal of plastic”. ID23*  *“Probably like, all the things about it going into the ocean, because that has gotten really big recently, so just all the initiatives that I have seen for people to reduce their single use plastic, because there are tons of things online on social media and everything for like the reusable straws, err, not using plastic bags, all those kinds of things, and definitely like, the waste in the ocean would be what I would think of first” ID22*  *“I think a big turning point in the US was all the turtles and so there were posters everywhere there were commercials every 5-minutes on TV and things like that that, was showing the rings that cans come in, like a six pack of cans we were saying this everywhere this was a couple of years ago but it was everywhere and I didn't care if it was very hard to see they were showing things that were really gruesome, and that was the turning point for a lot of my friends and I, because you're finally seeing it, it wasn't just plastic on a beach, which is sad, but you're seeing something else suffering because of it, and you feel the impact more, because I mean any kind of living thing you don't want to hurt them, well, laughing most people don't want to hurt them, but definitely the animals, that hits home more especially for me”. ID22*  *“I think about the packaging that I eat my lunch from but also like also water bottles.*  *Yes, and it's just like those commercials about like plastic getting into the fish and stuff and birds picking it up unfortunately and its very harmful for them. That also comes to mind”.ID25* |

| **Opportunity** |
| --- |
| There is a need to make recycling easier by improving labelling on food packaging  *“If there is a label that says “this is recyclable”, and that is the most obvious thing because if we buy it, we obviously look at the things and so if there is a big sticker that says recyclable then that would be good”. ID19*  *“I do think the two arrows that are on it at the minute, the chunky arrows, I take that as this can definitely be recycled, but then on fruit punnets there is that kind of triangle of arrows which I take to mean it can be recycled in some areas, but I don't know if that is right, and I would generally think, it’s probably unlikely that this is recyclable in my area, black bin, so I feel that specific kind of triangle of arrows doesn’t work, it’s too ambiguous”. ID8*  *It’s usually in very small print, which is easier to read once you have got it out of the packet, once you are home, and so what would help is not whether it is recyclable or not, as that is shown, but in reminding me when shopping to think about it, could just be a larger more obvious symbol because then when you are picking it up, whatever it is, err, so you are confronted with that at the point of purchase, rather than at home”. ID4*  *“There are certain things that you get and the symbols on the bottom of them aren’t really clear and so you can’t tell if they are recyclable. I had a packet of crisps the other day, and it had the recycling sign on it then underneath it, it said, in tiny writing, this packaging is not available for recycling at the moment, and I was, like, but the recycling sign is on it (laugh)and so anyone looking at it with a quick glance will throw it into the blue bin, but it won’s be recycled because it can’t be now, and then sometimes you get an instant meal, you can’t really tell, sometimes they would say check your local recycling but I don’t know if you look on line, if they do tell you exactly”. ID20*  *“It is always this small little thing in the corner of the bottle, that nobody really sees It’s usually like, this transparent thing on the bottom, it’s usually this one, it says things like numbers like 1,3,5 or 7, it's not really telling you whether it's recyclable or not. I don't see any reason for people putting that on, because people are not going to pay attention to it”.ID19*  *“This doesn't have anything on it (sandwich pack) hence it just makes me believe that I’m meant to throw everything out. Yeah, because obviously I don't know the exact composition of what went into this box, so I'm thinking, I'm doing something good by just throwing everything into the recycling box. But yes, if they could make it more visible, and on every individual component, so this would have a little black thing on it, and this would have a little black thing on it”. ID25*  *“It’s not clear at the minute and there are a lot of symbols and crazy instructions and things, that people feel ok about buying this because it can be recycled but the fact or that it can’t very easily be recycled so I think that needs to be an awful lot more clarity in the market on some packaging”. ID26*  *“The (supermarket) meat packaging can be a dark green colour, sometimes, or dark brown, that kind of thing, so maybe some sort of stamp that can make it clearer, or clearish, but with some sort of stamp that marks it out as different. You’d probably want that anyway to help the consumer, so that they would know it was authentically plant based authentically biodegradable so sort of a dual purpose”. ID10*  There is a need to make recycling easier by improving labelling on food packaging (cont).  *“I would be quite confident that I can recycle a yogurt pot or a tin or like a can from like a tin of beans and that type of thing or like beer bottles, the things that get me are when like you’ve got combinations of materials, like something with a film lid, like a plastic container with a film lid and like if you’ve already taken the outer packaging off something and you have thrown that away and you have kept the rest of it and you don’t have the little film can’t be recycled but the carton can, so yeah, those things I think in general are really the only ones which cause me any confusion (laughing)”. ID3*  *“Yeah, symbol or a colour. I would say a colour yes, something that is easy on the eye so that it is quick and easy for people to understand the differences between the categories of items, personally I think that would be more useful”. ID1*  *“I think that, you know, you have a lot of products already that can be recycled, but I think the sign on them really isn’t that salient at all, in fact it is really easy to ignore it, so I think something visual in terms of colour, you know almost in the same way that food packaging now has to have a breakdown of you know, what percentage of the product contains salt, er, that kind of colour coded thing, I think something along those lines that makes it easy to understand whether or not the product can be recycled”. ID1*  *“Yeah because I do think that people on the whole are fairly visual creatures and if you can make it easily visual for them so that you don’t have to think about something because obviously glass and plastic they all have different textures to them they feel different so people know the difference between those two. But visually though it would make it just that little bit easier than that and if it was standardized, it would be even better and people could latch on to that and symbolically of course if you go with the green, it would attract a certain number of people, black tends to turn people off when it comes to these sorts of things, so they could say ok black is bad and the clear could be recyclable. Black could be - you’re killing the environment kind of thing, or red I guess may work in that instance. Green obviously has been marketed very well in the last 40 years at this point, and so it has been well established if you see something that is greenish, it has that immediate connotation, whether it's true or not, of being environmentally friendly, good for the earth, “natural”. ID10*  *“The government has done things like, kids’ food you’ve got like a traffic light kind of thing, you know, where it says, you know, green, you can eat as much of this as you want… and it’s almost like there needs to be something as straightforward as that, that says, you know, this is fine, you can buy these things and it can be like plant based plastics, or biodegradable plastics and then these are things you shouldn’t really buy unless it’s necessary, as it will just, and if you must buy or have to buy you should chuck into recycling if you can, but I think there is just not enough that level of clarity around all of that”. ID12*  *“I think that, you know, you have a lot of products already that can be recycled, but I think the sign on them really isn’t that salient at all, in fact it is really easy to ignore it, so I think something visual in terms of colour, you know almost in the same way that food packaging now has to have a breakdown of you know, what percentage of the product contains salt, err, that kind of colour coded thing, I think something along those lines that makes it easy to understand whether or not the product can be recycled. A symbol or a colour. I would say a colour yes, something that is easy on the eye so that it is quick and easy for people to understand the differences between the categories of items, personally I think that would be more useful”. ID1*  *“It could be improved by, I know it normally ends up as a design issue, because I am only realising now, whenever I read this, I have to infer, I have to check locally for recycling information because it doesn't actually say, but it's more of like a heuristic from, I know what I am looking for, because the issue in and of itself might be, that this only helps people who are looking for recycling information, and doesn't act as a gateway to encouraging people to recycle things properly, and even then it does provide very little information check locally”. ID23* |

| Visual cues adding to confusion  *“It’s not clear at the minute and there are a lot of symbols and crazy instructions and things, that people feel ok about buying this because it can be recycled but the fact or that it can’t very easily be recycled so I think that needs to be an awful lot more clarity in the market on some packaging”. ID26*  *“… the triangles still confuse everybody, and nobody looks at the triangles, in the recycling world nobody looks at the triangles, it’s tubs, pots and trays. But the householder still sees triangles as the be-all and end-all and it has to be, if it says is recyclable then it has to be, so it goes in the bin, but it’s not”. ID32*  *“It is always this small little thing in the corner of the bottle, that nobody really sees. It’s usually like, this transparent thing on the bottom, it’s usually this one, it says things like numbers like 1,3,5 or 7, it's not really telling you whether it's recyclable or not. I don't see any reason for people putting that on, because people are not going to pay attention to it”. ID19*  *“…but I do think the two arrows that are on it at the minute, the chunky arrows, I take that as this can definitely be recycled, but then on fruit punnets there is that kind of triangle of arrows which I take to mean it can be recycled in some areas but I don't know if that is right, and I would generally think, it’s probably unlikely that this is recyclable in my area, black bin, so I feel that specific kind of triangle of arrows doesn’t work, it’s too ambiguous”. ID8* |
| --- |
| Removing perceived physical obstacles will encourage more recycling  *“It's not the people that want plastic packaged things, I feel like, it's not an act of choice they are just taking what is there in front of them, and I don't think anyone would really endorse saying, I want the packaged product, it is just all down to convenience, and the options they are given I think”. ID8*  *“Yes we do [have a food caddy], and it was used for a while, until we ran out of the little bin liners, because you have to get the special size, or the ones that decompose, and they haven't been bought since. If there were the little bin liners we would still be using that caddy, for sure. Even though I think the bin men leave them off. And it’s also because the caddies are very small, and generally once they were full it would be end of the food recycling then for that week.” ID8*  *“Putting big stickers on the bins that really tell you what you meant to recycle and give us even more bins, for more different types of plastics. I used to live in Poland when I was very little and I remember seeing, there was never just one recycling bin, it was always like, glass, paper, plastic and I feel like if people had more bins, they would know, so if they throw stuff out and because if you see glass and you see paper, you wouldn’t throw glass into the paper bin. I know they are both recyclable but, you get the gist of what I am saying”.ID25*  *“We had missed one of the bin days, so we had some of those recyclable bin bags and we put some of our recycled stuff in and set them out but they just left it there because it wasn’t in the boxes but we didn’t realise so, we had to go to the recycling somewhere and that was a bit annoying, but like, for four people in a house, one of those fills pretty quickly”. ID20*  *“I think the new system with the stackable bins, I think that’s good and that has encouraged, but it’s not where I live, it’s only in certain parts of town. I also think that all the council's in NI need to get together and create one system and think it's really annoying that in some places you can recycle your glass, and some places you can't, and I think we would catch more materials in this segregated, because it's really good fun and people enjoy popping things into different bins, I think that is quite interesting and I think if you look at places like Sweden or Denmark, or Norway, I don't know which one, but they have 7 different recycling bins and I think that that makes it really easy I think printing glossy leaflets about recycling is hypocritical and frustrating”.Id24* |
| Removing perceived physical obstacles will encourage more recycling  *“Yeah, like, I have no idea where the closest recycling centre is, but I’m sure it’s not within walking distance, or even just have a main blue bin, a big wheelie bin that would make things easier and you could have the boxes as well, and if you did overflow, you could put it into the bin and they could lift it that way but they probably won’t want overflows for the small bins, then I just think it works better, like we never fill our bin at home the big blue bin, but if it was smaller ones they just take up more room, and have less space, at the same time”.ID20* |
| There is an expectancy that others in power should take action  *“A lot of it is educating people and so I think the council should do more in that area they should hold more workshops, zero waste workshops and showing people, and making them feel more empowered. Because they don’t at the minute”. ID17* |
| Manufacturers need to be producing less plastic  *“I think we should be going along the lines of encouraging people to come up with the better packaging product rather than coming up with a solution at the end to what do we do with it”. ID18*  *“Everyone has a personal responsibility, obviously I have to believe that this is my practice as a personal responsibility for their own environmental practices but much like tracing everything to the sources of the issue organisations in their environmental practices, for example, like in how they go about manufacturing products, whether they decide to use sustainable plastics, alternative plastics, like plastics or materials made out of biomass, and also the design of the products ,it's really, there's a lot of onus on the companies, to design products in a way that facilitates good environmental behaviour”. ID23*  *“I just find it frustrating because I just think it’s unnecessary and I think that a) why does this stuff have to end up in the sea, or at the side of the road, so there’s that, and then b) why is some of this stuff actually still being made and purchased in these quantities when it’s perfectly possible to get away , perfectly possible to live without producing some of the stuff in the first place so yeah, I just find it, it’s a frustrating situation but one which seems to be getting kind of worse probably, than better I think”. ID12*  *“I think that sort of stuff needs to be regulated by the government, and I think it's great that people are using their initiative and trying to make a difference and I applaud all the small companies who are doing it, but I think now that we are getting into this area where there could be like 90 different types of compostable cup if not all those compostable cups are compostable in (city) centre in our waste disposal then they should not be used”. ID24* |

| Governments can intervene at a number of levels to address plastic waste problem  *“I still think not enough is done by individual council's to make people aware of the importance of recycling, and definitely not enough is done to make people aware of how to recycle properly, and it's something that is obviously is in all the council's interests that they really should be putting more money into this because if you don't change the way people think about recycling how are you going to get people more likely to recycle”. ID1*  *“I mean at a very, a lay perspective and from a distance, I think there’s been an argument for why bottle returning things won’t work, but it seems to be to be a good idea, it could be a load of rubbish, but it seems to me to be a good idea, I’m not an expert, and there are things like that, that would be encouraging people, I also think that actually, one of the things I’ve seen, which never used to be there is ah, water fountains, you are seeing much more of them and so you now see water fountains at the airports, which I think you never used to ID12*  *“I think it’s kind of poor branding to say that we don't actually care about the environment, we just want you to buy this, but we don't care what you do with this packaging afterwards, so we have made totally unrecyclable, yeah, I think they should be held accountable, like government's held Tesco’s and Sainsbury’s accountable for their plastic bag use and like imposed that levy onto the consumer but it was through the companies that were manufacturing their plastic bags and stuff and you now don’t get those single use, do you know like those flimsy plastic bags you get those bags for life but you don’t the single use ones”. ID3* |
| --- |
| There is a desire to see retailers providing less disposable plastic; and more ecologically friendly food/drink containers  *“But it also comes back to fast food or takeaway, or sandwich places, I think all of their products should be based on recyclable and there should be one bin once you have finished eating whether it's a container or a fork, a wrap, it should all be put in the one bin, and the bin should be a compostable bag where it’s all taken away”. ID18*  *“I think they do need to make some changes in the law, you know X [retailer] their coffee cups, I have my own reusable cup anyway, but I noticed they're using Veg-ware so they are compostable and I think will it's possible to do that, so why can't other chains do that as well”. ID26.*  *“I believe that has been shown from my personal experience and in other types of products, for example cleaning supplies, which claim they are environmentally friendly, for example, I am willing to pay a bit of a premium for those verses, something that goes down the drain and kills 1000 dolphins, kind of thing. So it seems to me that there is certain technology out there that is available, or could be developed, if the right incentives were in place”. ID10*  *“I was buying some coffee the other day, beans, coffee beans and it was in a kind of, you know, one of those foil kind of bags that coffee beans come in, and then it had a paper kind of surround which is just a branding thing and I just felt, I just thought, that is just completely… so I didn’t buy that, so, you kind of start to make some choices about things you cannot buy, and you think actually, that’s just pathetic, so you don’t buy it”. ID12*  There is a desire to see retailers providing less disposable plastic; and more ecologically friendly food/drink containers (cont.)  *“I think it’s more like, if there was an alternative, err, you know like shower gel as a good example actually I have just stopped buying, we’ve stopped buying shower gel and we’d just use soap, and even if the shower gel bottle said ok, this is in a plant based bottle, which is biodegradable, I’d probably look at it and think but, you know, this bar of soap is wrapped in a single sheet of paper almost and so, I suspect, I don’t know, but I suspect probably, err I don’t, you know now I’ve probably changed my, and I don’t think it’s necessary to buy that because there’s probably even less packaging with a bar of soap”. ID12* |

| **Motivation** |
| --- |
| Personal motivation to recycle could be activated by making the plastic waste problem visual and close to home  *“If that was right next to the entrance to your housing estate there was this huge big landfill, just like stinking and sitting there, like everywhere you know what I mean. We just put it all to the back of our minds and I think we do that with everything, like we ignore things that aren't right in front of us, and like it's just like that kind of chosen ignorance I think is a big part of it”. ID3*  *“The visual aspect really works in that case - it does work for me. We can go back to that quote that “one person's death is a tragedy, but that a million is just a statistic”. It's also why I think film and any kind of creative art is, when it is effective, is effective because it takes the personal and makes it universal”.* *ID10*  “*So putting big stickers on the bins that really tell you what you meant to recycle and give us even more bins, for more different types of plastics”. ID25*  *“But it's the personal impact of a person's actions doesn't have the reciprocal effect on them, that it should. I think that people whenever they see immediate feedback on their actions are much more likely to consider the consequences than if they just ship it off for someone else to deal with” ID10.*  *“I suppose you see a Coca-Cola bottle or whatever else it could be, and I know that I've drank quite a few of those, so how do I know that that is not my bottle? I think that's the thing that scares me and I think it does feel, it's those things that make it feel personal, even though it is so far removed, and we don't have these turtles or dolphins near Ireland, but we still want to look after them”.ID24*  *“I’m interested in the environment and do a lot of hill walking and stuff, and often if I find stuff, rubbish, I usually bring it back, so like bottles, and I would come back with 2 or 3 plastic bottles from the hills, something like that, or rubbish, or if I was to go to a layby where I would go walking and there is stuff there, I would pick bits up and chuck it in the back of the car and then I can chuck it in the bin”. ID12*  *“I feel, if I was prompted to think about it, or I had just seen a video, like maybe on Facebook scrolling, and something about waste came up before I went shopping, absolutely. But it kind of just goes out of your head when you come to do your daily shop.” ID8* |
| The emotional impact of seeing pollution damaging wildlife, can act as emotional nudges towards reducing plastic waste  *“The animal part touches me, because I'm a vegetarian and I eat a mostly vegan, and it's definitely very sad to see the animals being harmed… I didn't care if it was very hard to see they were showing things that were really gruesome, and that was the turning point for a lot of my friends and I, because you're finally seeing it, it wasn't just plastic on a beach, which is sad, but you're seeing something else suffering because of it, and you feel the impact more, because I mean any kind of living thing you don't want to hurt them, well, most people don't want to hurt them, but definitely the animals, that hits home more especially for me”. ID22*  The emotional impact of seeing pollution damaging wildlife, can act as emotional nudges towards reducing plastic waste (cont.)  *“I just, I suppose you see a [plastic] bottle or whatever else it could be, and I know that I've drank quite a few of those, so how do I know that that is not my bottle? I think that's the thing that scares me and I think it does feel, it's those things that make it feel personal, even though it is so far removed, and we don't have these turtles or dolphins near Ireland, but we still want to look after them, and yes, it's hard to see those images it makes you want to stop and save the animals”. ID24*  *“I actually read an article about the typhoon of plastic that is in the oceans at the moment, and how many issues this causes, particularly as well with the highlighted damage of plastic straws, for sea life all these stories together have sort of really highlighted plastic as a negative environmental thing and that is why when I'm thinking about plastic, the first thing I normally think about is all these stories about how animals are being harmed by the production of and wasting of plastic or I should say the incorrect disposal of plastic”. ID23*  *“Yes I think the worrying thing is that they have discovered now about, these titsy witsy little bits of plastic in fish that we eat, and we are used to thinking it was healthy eating fish, and now we could be poisoning ourselves with what is the build-up of the residue.”ID7* |
| Even those who perceive themselves to be eco-conscious are limited in what they are willing to sacrifice  *“Yeah, I feel like with clothes it is a lot more invisible and it is not talked about nearly as much and I think I am not informed at all about plastic in clothes. And definitely I think I've got a kind of lot more cognitive dissonance there, about going to (shop name) and just really pushing that out of my mind and I think that when it comes to fashion I do nothing to be environmentally friendly when I think about it.. people have been talking about recycling for so long and is it just going to take a while to catch up”. ID8*  *“So I tend to go to greengrocers and stuff to buy produce, because it's generally not wrapped there because even in the supermarkets, the celeriac, the supermarkets put masses of cellophane around it. If I needed something I would still buy it, feeling very guilty about it, but there is very little I have found that I actually need in plastic these day”. ID26*  *“And I think. you know, this definitely, I know myself, if I really put more thought into it, I could definitely source products that are more environmentally friendly in terms of the packaging, because of the packaging. But just because I'm very probably very lazy (short laugh), I just go for the products in the shops that I know”. ID1*  *“If you had a choice between, and you had to buy some milk and I'm one carton it says it's made from plant based this plastic comes from plants or this plastic comes from oil, which one would you prefer?” “The plants”. Interviewer- “Would you pay a bit more for it do you think? I mean how much would you pay, if it was £1 would you be prepared to pay 10p more up to 50p more is there a level at which you would say, no that's too much?”. “Probably the 50p that will be the absolute max, because your basic basically paying another 50% for it”. ID24.*  *“If I knew that one of them didn’t have oil in it I would obviously get the one that would biodegrade but that would make me think like, wouldn’t those ones like way more expensive than normal plastics since big companies they make so much plastic that's it cheap for them to produce but I feel like for a while you know ethical plastic would be super expensive and that would discourage people from buying it”. ID25*  Even those who perceive themselves to be eco-conscious are limited in what they are willing to sacrifice (cont.)  *“But now I would probably spend a little bit more, but if it was, the difference of a £1.40 per bottle versus £1 per bottle, I don't think I could get over that, even though it's 40p it's 40% of the price of the alternative”. ID23*  *“I grew up in the (Region) which tends to be one of the most progressive parts of the of (country) and very much in the forefront of the environmental movement the organic movement and I went to an undergraduate University which was very left wing, environmental, radical, that kind of thing, but I also worked for a couple of years for an organic garden store. So I've been pretty much indoctrinated from a young age into the belief set, it is important to take care of the planet of the environment and so, so yes I would say that most of it probably came from a very young age. I think I've got an older, the convenience of people maybe the government, making it easy for me to do the right thing, makes a huge difference to my willingness to do things”. ID10*  *“They have to be seen to be making some sort of response from public pressure that way, so I think everybody has their part to play. But I mean, we would still would buy the foods we need, we don't like the plastic wrapping, but it's not going to put us off buying a cucumber, you know, but we don’t like it.”. ID7*  *“Why do you think they just put it into the ordinary waste when there is an opportunity to recycle?” “Apart from laziness maybe they think they it's not going to make any difference, like one bottle is not going to save the planet, you know what I mean?”. ID25*  *“When it is thinking about marine life it’s a different kind of thing, in the sense that you don’t have the risk there, since people don’t keep marine life in their own back garden generally speaking, and they just think, oh, I’ve just done my bit for this one little thing. This seems to me to be the kind of issue that raises awareness more generally and will impact on their general consumption of plastic. Whereas with issues like the drinking straws, the risk is that more narrowly, people will campaign against those kinds of things, but people don’t take that as something that stands for a wider problem, they take it as something they can feel good about”. ID4* |

| **Motivation – Non conscious/automated** |
| --- |
| *“If strawberries come in like a very light punnet, or if they're all wrapped up and made to look very nice and presentable I think I would still go for the very nice and presentable ones. I would give up my ethics -unless I am consciously thinking about it of course.” ID23*  *“Yeah because I do think that people on the whole of fairly visual creatures and if you can make it easily visual for them so that you don’t have to think about something because obviously glass and plastic they all have different textures to them they feel different so people know the difference between those two. But visually though it wold make it just that little bit easier than that and if it was standardized, it would be even better and people could latch on to that and symbolically of course if you go with the green it would attract a certain number of people black tends to turn people off when it comes to these sorts of things, so they could say ok black is bad and the clear like you said could be recyclable black could be you’re killing the environment kind of thing (laughing) or red I guess may work in that instance.” ID10*  *“Because behind the scenes, I'm really positive about recycling, and I value it really highly, but actually, I don't think that really occurs to me when I'm standing in (supermarket name) and maybe it would be (supermarket name) and (supermarket name) for food shops”. ID8*  *“And also being environmentally friendly too, they see, for example my friend said don't do that because dot, dot, dot and all the information was there, but all the information was there, but I was not conscious, I was not awake but she told me this, this, and this and she showed me how to do it. So then I was ok, this is right, ok, I am going to change that”. ID1.*  *“I am conscious of environmental issues, but if someone wasn't conscious of environmental issues, and perhaps had never thought about them, and their first product that ever thought about recycling was just as they finished that pot of (product), that's really a barrier to them changing their behaviour because they won't know how, about how to change it in a successful manner”. ID23*  *“If strawberries come in, like a very light punnet, or if they're all wrapped up and made to look very nice and presentable, I think I would still go for the very nice and presentable ones, I would give up my ethics - unless I am consciously thinking about it of course”. ID1*  *“Well it’s not that I don’t know, it is when I’m shopping, at the shopping stage, it won’t enter my head to look closely at the plastic film which is what I am talking about, you know, the thin plastic film which is sometimes recyclable and sometimes not?”. ID4*  *“While in the supermarket, the different levels of decisions whenever I'm in the supermarket I wouldn’t ever think that deeply about it, I don't think I would make decisions primarily on the anticipation of the eating and preparing the food and not acknowledging packaging”. ID23*  *“So, it is me that’s doing the shopping in my house, and I guess mostly it probably comes down to price, I would say, which I don't like to say, because behind the scenes, I'm really positive about recycling, and I value it really highly, but actually, I don't think that really occurs to me when I'm standing in [the supermarket]”. ID7* |

| **Motivation – Non conscious/automated**  *“It is when I’m shopping, at the shopping stage, it won’t enter my head to look closely at the plastic film which is what I am talking about, you know, the thin plastic film which is sometimes recyclable and sometimes not? So once I get it home, err, so in the shop I am not thinking, and once I am home, as soon as I use the item and I need to chuck away the packaging, I’ll know once way or the other, because either it will say recyclable or it won’t”. ID4*  *“In terms of packaging, the kind of things I would buy have quite a short shelf life, and so I wouldn't necessarily pay a lot of attention to packaging…I think in terms of purchasing food, I would say it is about 95% about the actual product, and probably about 5% thinking about the packaging. I think maybe a few years ago, I probably did pay more attention”. ID1*  *“Once I get it home, err, so in the shop I am not thinking, and once I am home, as soon as I use the item and I need to chuck away the packaging, I’ll know once way or the other, because either it will say recyclable or it won’t. If it doesn’t, I know it isn’t. And mostly, it won’t be ambiguous it will say, not currently recyclable, so I don’t seek, err, you know I don’t go anywhere further to seek information about that, I just go with what’s on the packaging and if it doesn’t say it is recyclable, then I won’t recycle it, usually it will be tell me it’s not recyclable”. ID8* |
| --- |
