# Supplementary material for: “If it is not made easy for me, I will just not bother”. A qualitative exploration of the barriers and facilitators to recycling plastics
Source: PLoS One. 2022 May 3;17(5):e0267284. doi: 10.1371/journal.pone.0267284 (PMC9064103; doi:10.1371/journal.pone.0267284)
Supplement: Attachment — Submitted filename: PONE-D-21-36666.pdf [file pone.0267284.s003.pdf]

PLOS ONE

"If it is not made easy for me, I will just not bother". A Qualitative Exploration of the Barriers and Facilitators to Recycling Plastics

--Manuscript Draft--

**Manuscript Number:**	PONE-D-21-36666
**Article Type:**	Research Article
**Full Title:**	"If it is not made easy for me, I will just not bother". A Qualitative Exploration of the Barriers and Facilitators to Recycling Plastics
**Short Title:**	A Qualitative Exploration of the Barriers and Facilitators to Recycling Plastics
**Corresponding Author:**	Emma Berry<br>Queen's University Belfast EPS: Queen's University Belfast Faculty of Engineering and Physical Sciences<br>Belfast, UNITED KINGDOM
**Keywords:**	Plastic Waste, Recycling Behaviour, COM-B Model, Food Packaging, Environmental Conservation
**Abstract:**	Millions of tonnes of plastic are ending up in our oceans and creating a global environmental hazard. Around 50 percent of plastics are designed for single use, mainly for food packaging. Plastic waste is often found discarded as litter, despite significant investment over the last 20-30 years to increase recycling facilities and kerbside collection of waste materials. While there is a substantive body of literature about general recycling behaviour, few insights exist that may help us understand why a sizeable number of consumers and householders are failing to sort plastics for recycling at optimal levels. Consequently, used plastic is ending up in landfill and is polluting the natural world. This current study used in-depth qualitative interviews within a framework that seeks to identify underlying opportunities, motivations, and capabilities of individuals when it comes to plastic recycling. The principal findings are that the respondents are generally positive about recycling and express a degree of environmental concern about the damage plastic waste causes to the natural world. However, this concern wields only a small influence on consumers' efforts to obtain accurate information about plastics disposal. It carried even less weight when visual reminders of its harmful impact are absent. Thus, when faced with a confusing array of packaging that combines several types of plastics, recycling rates remain disappointingly low. Householders believe the responsibility for making plastic recycling easier rests heavily with local government and manufacturers. Until a greater sense of shared responsibility is nurtured and greater uniformity in packaging is achieved, plastics recycling rates will continue to fail to reach optimal levels.
**Order of Authors:**	Deborah Roy, Ph.D
	Martin Dempster, PhD
	Emma Berry, PhD
**Additional Information:**	
**Question**	**Response**
**Financial Disclosure**<br><br>Enter a financial disclosure statement that describes the sources of funding for the work included in this submission. Review the submission guidelines for detailed requirements. View published research articles from *PLOS ONE* for specific examples.<br><br>This statement is required for submission	This work was supported by the Engineering and Physical Sciences Research Council (ESPRC) Grant number EP/S025545/1.<br><br>https://epsrc.ukri.org/

Powered by Editorial Manager® and ProduXion Manager® from Aries Systems Corporation

and **will appear in the published article** if the submission is accepted. Please make sure it is accurate.<br><br>**Unfunded studies**<br>Enter: *The author(s) received no specific funding for this work.*<br><br>**Funded studies**<br>Enter a statement with the following details:<br>• Initials of the authors who received each award<br>• Grant numbers awarded to each author<br>• The full name of each funder<br>• URL of each funder website<br>• Did the sponsors or funders play any role in the study design, data collection and analysis, decision to publish, or preparation of the manuscript?<br>• **NO** - Include this sentence at the end of your statement: *The funders had no role in study design, data collection and analysis, decision to publish, or preparation of the manuscript.*<br>• **YES** - Specify the role(s) played.<br><br>* typeset	
**Competing Interests**<br><br>Use the instructions below to enter a competing interest statement for this submission. On behalf of all authors, disclose any competing interests that could be perceived to bias this work—acknowledging all financial support and any other relevant financial or non-financial competing interests.<br><br>This statement is required for submission and **will appear in the published article** if the submission is accepted. Please make sure it is accurate and that any funding sources listed in your Funding Information later in the submission form are also declared in your Financial Disclosure statement.<br><br>View published research articles from *PLOS ONE* for specific examples.	The authors have declared that no competing interests exist.

**NO authors have competing interests**<br><br>Enter: *The authors have declared that no competing interests exist.*<br><br>**Authors with competing interests**<br><br>Enter competing interest details beginning with this statement:<br><br>*I have read the journal's policy and the authors of this manuscript have the following competing interests: [insert competing interests here]*<br><br>* typeset	
**Ethics Statement**<br><br>Enter an ethics statement for this submission. This statement is required if the study involved:<br><br>• Human participants<br>• Human specimens or tissue<br>• Vertebrate animals or cephalopods<br>• Vertebrate embryos or tissues<br>• Field research<br><br>Write "N/A" if the submission does not require an ethics statement.<br><br>General guidance is provided below. Consult the submission guidelines for detailed instructions. **Make sure that all information entered here is included in the Methods section of the manuscript.**	Ethics approval was obtained through Queen's University Belfast prior to recruitment and written/typed informed consent was obtained from participants using electronic and physical consent forms.

Powered by Editorial Manager® and ProduXion Manager® from Aries Systems Corporation

**Format for specific study types**<br><br>**Human Subject Research (involving human participants and/or tissue)**<br>• Give the name of the institutional review board or ethics committee that approved the study<br>• Include the approval number and/or a statement indicating approval of this research<br>• Indicate the form of consent obtained (written/oral) or the reason that consent was not obtained (e.g. the data were analyzed anonymously)<br><br>**Animal Research (involving vertebrate animals, embryos or tissues)**<br>• Provide the name of the Institutional Animal Care and Use Committee (IACUC) or other relevant ethics board that reviewed the study protocol, and indicate whether they approved this research or granted a formal waiver of ethical approval<br>• Include an approval number if one was obtained<br>• If the study involved *non-human primates*, add *additional details* about animal welfare and steps taken to ameliorate suffering<br>• If anesthesia, euthanasia, or any kind of animal sacrifice is part of the study, include briefly which substances and/or methods were applied<br><br>**Field Research**<br><br>Include the following details if this study involves the collection of plant, animal, or other materials from a natural setting:<br>• Field permit number<br>• Name of the institution or relevant body that granted permission	
**Data Availability**<br><br>Authors are required to make all data underlying the findings described fully available, without restriction, and from the time of publication. PLOS allows rare exceptions to address legal and ethical concerns. See the PLOS Data Policy and FAQ for detailed information.	Yes - all data are fully available without restriction

A Data Availability Statement describing where the data can be found is required at submission. Your answers to this question constitute the Data Availability Statement and **will be published in the article**, if accepted.<br><br>**Important:** Stating 'data available on request from the author' is not sufficient. If your data are only available upon request, select 'No' for the first question and explain your exceptional situation in the text box.<br><br>Do the authors confirm that all data underlying the findings described in their manuscript are fully available without restriction?	
**Describe where the data may be found in full sentences. If you are copying our sample text, replace any instances of XXX with the appropriate details.**<br><br>• If the data are **held or will be held in a public repository**, include URLs, accession numbers or DOIs. If this information will only be available after acceptance, indicate this by ticking the box below. For example: *All XXX files are available from the XXX database (accession number(s) XXX, XXX.*).<br>• If the data are all contained **within the manuscript and/or Supporting Information files**, enter the following: *All relevant data are within the manuscript and its Supporting Information files.*<br>• If neither of these applies but you are able to provide **details of access elsewhere**, with or without limitations, please do so. For example:<br><br>*Data cannot be shared publicly because of [XXX]. Data are available from the XXX Institutional Data Access / Ethics Committee (contact via XXX) for researchers who meet the criteria for access to confidential data.*<br><br>*The data underlying the results presented in the study are available from (include the name of the third party*	Data Accessibility Statement: The dataset is held in a secure repository at Queens University Belfast Psychology Department. A Supplementary Data File containing a compilation of further supporting quotes are published with the manuscript. Interested researchers wishing further data should initially contact Professor Martin Dempster, at Queens University, Belfast.

and contact information or URL).<br>• This text is appropriate if the data are owned by a third party and authors do not have permission to share the data.<br><br>* typeset	
Additional data availability information:	Tick here if the URLs/accession numbers/DOIs will be available only after acceptance of the manuscript for publication so that we can ensure their inclusion before publication.

Powered by Editorial Manager® and ProduXion Manager® from Aries Systems Corporation

Manuscript

Click here to access/download;Manuscript;Manuscript -Final 23.11.2021.docx

Title: "If it is not made easy for me, I will just not bother". A Qualitative Exploration of the Barriers and Facilitators to Recycling Plastics

Authors and affiliations:

Dr Deborah Roy, Department of Psychology, University of Bath, Claverton Down, Bath, BA2 7AY dr321@bath.ac.uk

Dr Emma Berry*, School of Psychology, Queens University Belfast, Malone Road, Belfast, BT7 1NN e.berry@qub.ac.uk

Professor Martin Dempster, School of Psychology, Queens University Belfast, Malone Road, Belfast, BT7 1NN m.dempster@qub.ac.uk

Corresponding Author*: Dr Emma Berry; e.berry@qub.ac.uk

Abstract

Millions of tonnes of plastic are ending up in our oceans and creating a global environmental hazard. Around 50 percent of plastics are designed for single use, mainly for food packaging. Plastic waste is often found discarded as litter, despite significant investment over the last 20-30 years to increase recycling facilities and kerbside collection of waste materials. While there is a substantive body of literature about general recycling behaviour, few insights exist that may help us understand why a sizeable number of consumers and householders are failing to sort plastics for recycling at optimal levels. Consequently, used plastic is ending up in landfill and is polluting the natural world. This current study used in-depth qualitative interviews within a framework that seeks to identify underlying opportunities, motivations, and capabilities of individuals when it comes to plastic recycling. The principal findings are that the respondents are generally positive about recycling and express a degree of environmental concern about the damage plastic waste causes to the natural world. However, this concern wields only a small influence on consumers' efforts to obtain accurate information about plastics disposal. It carried even less weight when visual reminders of its harmful impact are absent. Thus, when faced with a confusing array of packaging that combines several types of plastics, recycling rates remain disappointingly low. Householders believe the responsibility for making plastic recycling easier rests heavily with local government and manufacturers. Until a greater sense of shared responsibility is nurtured and greater uniformity in packaging is achieved, plastics recycling rates will continue to fail to reach optimal levels.

Key words: Plastic Waste, Recycling Behaviour, COM-B Model, Food Packaging, Environmental Conservation

1 Introduction

Plastic materials, whose raw materials include crude oil, gas, etc. have become a valuable resource across the world [1] and used by many industries. Materials such as Polyvinyl chloride (PVS) are used to insulate homes, Polyethylene terephthalate (PET) is used to package food to keep it fresh, and Polypropylene (PE) is used to bottle water and drinks [2]. Never has their benefits been more visible than in the production of Protective Equipment (PPE) to prevent the spread of Covid-19. However, plastics were never intended to end up in our oceans, rivers and beaches and because of alarming levels of plastic pollution, plastic items have instead become a symbol of pollution and both human environmental harm [3].

One important action, supported globally, but more recently by America's leading plastic manufacturers [1] is to "*engage the entire plastics value chain, from plastic makers to brand companies, to all Americans*". The vision being, that plastics are routinely reused, and plastic waste is diverted from landfills and our oceans. In Europe, laudable goals have been set to increase the reuse of plastic waste, and yet the production of new or virgin plastics, far exceeds recycling rates [4].

An increase in the reuse of household plastic waste to make an important contribution to the circular economy, is predicated upon householders and consumers increasing existing recycling rates to optimal levels. Efforts to increase recycling behaviour will therefore need to draw on the latest research on human behavioural decision making around recycling, which spans the last 30-40 years. Most of this research has focused on understanding influences on human motivation, and when it comes taking action, and a dominant theory is the Theory of Planned Behaviour (TPB) [5,6]; which explains that behaviour intentions are influenced directly by favourable or unfavourable attitudes towards the behaviour, the extent to which one feels social pressure to engage in the desired behaviour, and how easy or difficult one perceives

the behaviour to be. Despite its widespread application, a major review of the use of TPB revealed that 86% of these studies neglect various indirect factors that can influence behaviours [7]. These factors include demographics [8-12]), rewards [10], feedback [11], recycling scheme design [13], scheme knowledge [14], environmental concern [15], antecedent behaviour [16], personal norms [17], and emotions [18,19].

The TPB model [5,6] is often applied without also measuring variables pertaining to the environmental context and any physical barriers which may exist, or the fact that waste disposal is part of our daily routines. If a behaviour is habitual, this, in of itself, becomes a barrier to any attempts to change that behaviour, because of the influence of environmental triggers or cues [20].

For example, very familiar signs can activate certain actions, with little thought going into the actual process [21], likewise, emotional responses to certain environmental stimuli can seamlessly drive or thwart certain actions [22]. The TPB, by placing an exclusive emphasis on psychological factors, consequently, falls short of being a comprehensive framework to explain recycling behaviour. We must therefore look to a more pluralistic framework, that considers situational factors as well as psychological factors. Such a framework is the Capability, Opportunity and Motivation (COM-B) model of behaviour [23,24], which captures all the factors important in a complex behaviour such as recycling. The COM-B model hypothesises that the interaction between three components, Capability, Opportunity and Motivation (COM), causes the performance (or not) of Behaviour (B). Capability refers to psychological and physical capability, where psychological capability is the capacity to engage in the thought processes necessary for the performance of the behaviour, and physical capability is the capacity to engage in the physical activity necessary for performance of the behaviour. Opportunity covers all those factors that lie outside the individual that make the behaviour

possible and can prompt it. It is divided into physical opportunity (the opportunities provided by the environment such as kerbside collection services and recycling bins) and social opportunity (the impact of the cultural context on our thoughts/beliefs). Motivation is divided into reflective and automatic motivation. Reflective motivation concerns our evaluations/beliefs that are relevant to the behaviour; automatic motivation refers to our emotions and impulses or habits, that are relevant to the behaviour and may be activated by visual cues.

Previous research on recycling behaviour has focused on the capability and motivation aspects of this model but has paid much less attention to the opportunity element. More specifically, we do not know the extent to which physical opportunity could impact on recycling behaviour and given this gap, we are still lacking a comprehensive understanding of the determinants of recycling behaviour. Physical opportunities, in the context of the COM-B model, could also refer to the nature/design of the material that ought to be recycled, the complexity of the behaviour required to recycle, and any support or prompts provided to encourage recycling. Furthermore, a recent review [25] highlighted that while there has been a considerable amount of literature dedicated to understanding waste management behaviour, recycling, and the motivations behind it, research has mainly focused on the recycling of general waste, and not plastics specifically. The limited studies which have looked at plastic, have focused mainly on drinks bottles and plastic bags [25] or at waste in our oceans [26]. In addition, with increasing availability of compostable/eco plastics, there is greater variability in the plastic products that individuals are presented with in day-to-day life, which can add to the confusion about what plastic materials are recyclable [27].

According to Garcia and Robertson (2017) [28], about half of the annual global production of solid plastics, or 150 million tonnes, is thrown away worldwide each year. This inappropriate and irresponsible disposal of potentially recyclable material must be urgently addressed because; the environment is harmed when plastics reside in landfill or enter our water courses [29]; it is an economic opportunity cost [28]; and recycling plastics to use again saves energy compared with producing virgin materials.

When recycling facilities are present, successful recycling of plastics will depend, to a large degree, on individual behaviour. Not only do individuals need to distinguish between general waste and recyclable waste, but they are also required to separate out their recyclable plastics, because removing plastics from mixed waste after collection is not cost effective [30]. Therefore, it is imperative that we understand all the determinants of plastics recycling behaviour if we are to reduce the unsustainable situation regarding plastics use and disposal.

The strength of using qualitative techniques is that it allows the researcher to gain insights to thought processes around plastic disposal, in the environmental context where the recycling is mostly taking place (e.g., in the home). It also enables the researcher to probe and test decision making, and degree to which associated psychological and situational factors interact.

This study aims to isolate and understand the capability, opportunity and motivation factors which may determine rates of recycling of plastics behaviour. This is an important step towards identifying behaviour change strategies that may increase recycling of plastic products and packaging.

Materials and Methods

Rationale

The detailed explanation of the theoretical framework (Capability, Opportunity and Motivation (COM-B) model of behaviour) is available elsewhere [23,24].

Study Design

This qualitative study adopted a semi-structured interview design which was deemed most appropriate to address this complex research question. Ethics approval was obtained through Queen's University Belfast prior to recruitment and written/typed informed consent was obtained from participants using electronic and physical consent forms. An online survey of 756 NI residents designed to examine perceptions about plastic use, and plastic food packaging, provided a useful reference point for this qualitative study. This data related particularly to opportunity and motivation components of the COM-B model and are referred to in the results section (see Table 2). This study is written in line with the Consolidated Criteria for Reporting Qualitative research (COREQ) guidelines to support the sound reporting of methods and findings [31].

Sample and Recruitment

Recruitment took place during Autumn 2019, using convenience sampling via university websites, social media (Twitter, Facebook etc.) and word of mouth. Local supermarkets and businesses were also asked to place the flyers in their shop windows. Interested participants made contact through the researcher's university email address. Potential participants were provided with an information sheet and consent form in advance and advised that interviews would be recorded. The interviews took place on university premises. Participants were reimbursed reasonable travel expenses. Before the interviews began, the participants were given the information sheet and offered the chance to ask questions. The participants were also advised that they could stop at any time during the interview. If they still wished to proceed, they completed the consent form. Age and sex of participants is displayed in Table 1.

Table:1 Demographic Breakdown of Interviewees by Age and Sex

	SEX	
**AGE**	Male	Female
**18-24**	1	4
**25-34**	3	5
**35-44**	2	1
**45+**		2

Data Collection

All the researchers involved in the coding and analysis (DR, MD, EB) were experienced in the use of qualitative methods and adopted a phenomenological approach [32]. Semi structured questions and the interview schedule were designed based on COM-B theoretical domains. The semi-structured interview schedule included example scenarios to elicit current behaviours, beliefs, attitudes, and feelings towards a range of plastic products across familiar, everyday contexts. DR conducted the interviews and is an experienced qualitative researcher with a phenomenological orientation, but who also integrates this naturalistic enquiry with a realist stance [33]. This is because of her experience and knowledge of applying socio-cognitive models to explain sustainable behaviour and attitudes towards the environment. Pre-existing assumptions were acknowledged as potentially influencing interpretation of findings due to the subjective nature of this methodology, however, biased interpretation was mitigated by members checking of codes and regular team meetings to discuss the analysis [34]. The interviews lasted 45 minutes on average, and these were audio recorded. Three members of staff from the department participated, but otherwise, the participants were unknown to DR

before the interviews took place. The research team (DR, MD, EB) held coding meetings regularly, and concluded, after 18 interviews, that no new themes of note were emerging and consequently were satisfied that saturation point had been reached [35].

Ethics

Data analysis

The analysis was based on a semi-directed content analysis approach [36] along with a search for emerging themes. The researchers therefore allowed for the possibility of novel patterns to emerge while also considering the findings within the context of the COM-B framework, and other relevant theoretical frameworks. This also preserved the flexibility to offer interpretations of all the data, and to allow themes to be derived from the data. DR initially familiarised herself with the data and used NVivo 12 software to assist with coding by category and consistency, and then looked for any patterns that presented. This was an iterative process. A reflexive record was kept of the decision-making of the researcher as she coded and searched for patterns in the data.

Trustworthiness and data quality

A subset of the transcripts was coded by other members of the team (MD and EB) to ensure reliability and to ensure that findings were trustworthy and practically sound. Overarching themes were finally identified and checked by the research team to confirm they provided a good representation of the findings [36]. This process ensured that reflexivity was maintained throughout the analysis to support credibility and confirmability, particularly with recognition of the potential influence of researcher bias [34].

Results

A total of 18 participants were interviewed (see Table 1 for demographics). The themes extracted from the data are set within the context of the capabilities, opportunities, and motivations system (the 'COM-B' system [23,24,38]. Additional supporting quotes are

provided in the 'supporting information' file. However, naturally there was a certain degree of conceptual overlap in the topics that participants raised. The overarching themes, framed by the COM-B constructs, were as follows: 1) Capability (psychological): 1a) 'Confusion about what plastic can be recycled'; 1b) 'Lack of information-seeking behaviour to reduce uncertainty'. 2) Opportunity: 2a) 'The need to improve labelling on food packaging'; 2b) 'Visual cues in current designs add to confusion'; 2c) 'Reducing perceived physical obstacles encourages recycling'; 2d) 'Expectancy that others should take action'. 3) Motivation: 3a) 'Activating personal motivation to recycle by increasing the visibility of the plastic waste problem'; 3b) 'Witnessing pollution damaging wildlife is an emotional nudge to reducing plastic waste'; 3c) 'There are limits to what individuals are willing to sacrifice, even among the eco-conscious'; 3d) 'Community level engagement can endorse recycling behaviours'; 3e) 'Acting on impulse'. The content coding frequency analysis can be viewed in Figure 1.

Figure 1: A breakdown of the coding content relating to each domain, using Nvivo - according to COM-B Framework

[insert figure here]

Capability-Psychological

Confusion about what plastic can be recycled

According to Passafaro & Stefano (2017) [39], people must be able to recognise the recyclable materials, which bin they should be placed in, and know where and how to find additional guidance when needed. A considerable barrier to recycling identified among participants, was confusion about what can be recycled, as an inaccurate understanding emerged about plastic materials themselves. This confusion was compounded when participants were confronted with packaging that combined several types of plastic, or other materials such as paper.

"I would be quite confident that I can recycle a yogurt pot or a tin or like a can from like a tin of beans and that type of thing or like beer bottles, the things that get me, are when you've got combinations of materials, like something with a film lid, like a plastic container with a film lid". ID3.

"*I would say, and obviously there is so much plastic that food comes in, that you're not sure, and what I would, what I tend to do, is to put it all in the recycling bin and kind of think oh well, if it can't be recycled, then it's somebody else's problem - which is maybe not the best attitude to have" ID1*

"Yeah, so soft plastics I would be confused, about whether you can or can't cycle, and my take was that you can't and things like your punnets for fruit, and yoghurt pots, I would be unsure about, and they would go in the black bin" ID8.

Lack of information-seeking behaviour, to reduce uncertainty

Vining and Ebreo (1990) [40] found that knowledge is a significant predictor of recycling behaviour. But rather than seeking information from local authorities and recycling companies to educate themselves and aid decision making, our respondents instead appeared to routinely rely upon heuristics and routine.

> *"I have to infer, I have to check locally for recycling information because it doesn't actually say, but it's more of like a heuristic - when tired, I would never impose upon myself to go out of my way to find out more information about recycling or go to more effort. When I have individual resources depleted, like, I am tired, or things are not going well for me, if it is not made easy for me, I will just not bother". ID23.*

> *"I think the limit of seeking out information is like reading the side of the packaging. 'Because, like, it's that thing where normally if you are, say cooking, you're just throwing something in the bin - it's like, second nature". ID3*

Opportunity

The need to improve labelling on food packaging

Participants were asked specifically about the design of food packaging and the extent to which that helped or hindered recycling. Simple words written on the side of packaging are preferred by many; words that are clear, easy to locate and stand out, indicating whether the packaging can be recycled or not. Phrases such as "check locally" without any further details, were not considered to be helpful.

"If there is a label that says "this is recyclable", and that is the most obvious thing because if we buy it, we obviously look at the things, and so if there is a big sticker that says recyclable then that would be good". ID19

"It's not clear at the minute and there are a lot of symbols and crazy instructions and things, that people feel ok about buying this because it can be recycled but the fact is that it can't easily be recycled, so I think there needs to be an awful lot more clarity in the market on some packaging". ID26

The use of specific salient colours was also quite a popular suggestion for signalling that a package can be recycled, and this would make recycling decisions easier.

"I do think that people on the whole are fairly visual creatures and if you can make it easily visual for them so that you don't have to think about something because obviously glass and plastic, they all have different textures to them, they feel different so people know the difference between those two. But visually though it would make it just that little bit easier than that and if it was standardized, it would be even better and people could latch on to that and symbolically of course if you go with the green, it would attract a certain number of people… Green obviously has been marketed very well in the last 40 years at this point, and so it has been well established if you see something that is greenish, it has that immediate connotation, whether it's true or not, of being environmentally friendly, good for the earth, "natural". ID10

"The government has done things like, kids' food you've got like a traffic light kind of thing, you know, where it says, you know, green, you can eat as much of this as you

want… and it's almost like there needs to be something as straightforward as that, that says, you know, this is fine, you can buy these things and it can be like plant based plastics, or biodegradable plastics and then these are things you shouldn't really buy unless it's necessary, as it will just, and if you must buy or have to buy you should chuck into recycling if you can, but I think there is just not enough that level of clarity around all of that". ID12

"I think that, you know, you have a lot of products already that can be recycled, but I think the sign on them really isn't that salient at all, in fact it is really easy to ignore it, so I think something visual in terms of colour, you know almost in the same way that food packaging now has to have a breakdown of you know, what percentage of the product contains salt, err, that kind of colour coded thing". ID1

'Visual cues in current designs add to confusion'

The triangle symbol in general causes confusion, only two respondents understood its function.

"It is always this small little thing in the corner of the bottle, that nobody really sees. It's usually like, this transparent thing on the bottom, it's usually this one, it says things like numbers like 1,3,5 or 7, it's not really telling you whether it's recyclable or not. I don't see any reason for people putting that on, because people are not going to pay attention to it". ID19

"…but I do think the two arrows that are on it at the minute, the chunky arrows, I take that as this can definitely be recycled, but then on fruit punnets there is that kind of

triangle of arrows which I take to mean it can be recycled in some areas but I don't know if that is right, and I would generally think, it's probably unlikely that this is recyclable in my area, so I feel that specific kind of triangle of arrows doesn't work, it's too ambiguous". ID8

Recycling bins are provided in colours to indicate to consumers what materials should be placed in them but there is no standardisation of bins across the country. Participants highlighted the fact that different coloured bins are used by different local authorities (Councils) and that different materials can be recycled in these different bins. They reported annoyance with this changing system and that it would be easier for householders if the recycling system was standardised.

"It varies from Council area to Council area which I think…, is just crazy, why there can't be just a unified approach". ID21

"I think it's really annoying that in some places you can recycle your glass, and some places you can't, and I think we would catch more materials in this segregated [referring to stackable recycling boxes]". ID24

Some participants suggested that, in the absence of a standardised system, there should at least be clear information about which bin was used for which materials and that this information should be obvious at the point of recycling, rather than requiring the person to find the information on a website.

"If the information isn't presented to me at the forefront, when I need it, I just throw that into the black bin, the black bin being the general waste bin". ID13

"So putting big stickers on the bins that really tell you what you are meant to recycle and give us even more bins, for more different types of plastics". ID25

"Whenever I get home, I've been working all day, I've got the dinner to put on, got the kids to get sorted, home work to do, I really don't want to be standing at my recycling bin going, can this go in, I know that can't go in, you want it to be as relatively straightforward as you can, otherwise it's just becomes another complication in your life, and you are already working with time constraints". ID21

Reducing perceived physical obstacles encourages recycling

Some participants identified physical barriers to recycling that resulted in materials not being recycled appropriately. The obstacles identified by participants suggest that any inconvenience could be a demotivating factor that will influence behaviour.

"Yes we do [have a food caddy], and it was used for a while, until we ran out of the little bin liners, because you have to get the special size, or the ones that decompose, and they haven't been bought since. If there were the little bin liners we would still be using that caddy, for sure." ID8

"We had missed one of the bin days, so we had some of those recyclable bin bags and we put some of our recycled stuff in and set them out but they just left it there because it wasn't in the boxes but we didn't realise so we had to go to the recycling somewhere

and that was a bit annoying, but like, for four people in a house, one of those fills pretty quickly". ID20

Expectancy that others should take action

Third parties (government and business) were blamed for not doing more to encourage recycling. This resulted in a paradox in the responses, where more government regulation and less plastic manufacturing was advocated, hence removing choice, but more choice of products provided by retailers was also supported. The unifying element of this paradox is that participants were expecting action by perceived powerful others that would cause a change in their behaviour.

Manufacturers of plastic products were perceived to have some responsibility for any problems caused by plastic pollution. Participants suggested that manufacturers should produce less plastic or find alternative forms of plastic, and, in some cases, this was seen to be more effective than individual behaviour to deal with plastic pollution.

"I think we should be going along the lines of encouraging people to come up with the better packaging product rather than coming up with a solution at the end to what do we do with it". ID18

"I just find it frustrating because I just think it's unnecessary and I think that a) why does this stuff have to end up in the sea, or at the side of the road, so there's that, and then b) why is some of this stuff actually still being made and purchased in these quantities when it's perfectly possible to get away , perfectly possible to live without producing some of the stuff in the first place so yeah, I just find it, it's a frustrating

situation but one which seems to be getting kind of worse probably, than better I think". ID12

Local and central government were seen to have several roles in managing plastic consumption and recycling. These were: Educating citizens about recycling; rewarding people to motivate recycling; introducing a levy or ban on plastic products to discourage plastic use or encourage re-use, and influencing retailers' use of plastics.

"I still think not enough is done by individual council's to make people aware of the importance of recycling, and definitely not enough is done to make people aware of how to recycle properly, and it's something that is obviously is in all the council's interests that they really should be putting more money into this because if you don't change the way people think about recycling how are you going to get people more likely to recycle". ID1

"It would be good for central government to legislate for the big supermarkets not to be using, to cut down on the amount of plastic packaging that they use." ID13

Participants suggested that retailers could assist the recycling effort by using recyclable materials more frequently, providing bins for recyclable materials and not encouraging people to buy plastic by offering cheap plastic products.

"But it also comes back to fast food or takeaway, or sandwich places, I think all of their products should be based on recyclable and there should be one bin once you

have finished eating whether it's a container or a fork, a wrap, it should all be put in the one bin, and the bin should be a compostable bag where it's all taken away". ID18

"I think they do need to make some changes in the law, you know [retailer] their coffee cups, I have my own reusable cup anyway, but I noticed they're using Veg-ware so they are compostable, and I think will it's possible to do that, so why can't other chains do that as well". ID26.

Motivation

Activating personal motivation to recycle by increasing the visibility of the plastic waste problem

The impact of visual information was identified as a powerful way of encouraging people to engage more in recycling behaviour. Indeed, the absence of visual indicators of the consequences of not recycling was suggested as a reason why people do not engage in recycling to a greater extent.

"If that was right next to the entrance to your housing estate there was this huge, big landfill, just like stinking and sitting there, like everywhere you know what I mean. We just put it all to the back of our minds and I think we do that with everything, like we ignore things that aren't right in front of us, and like it's just like that kind of chosen ignorance I think is a big part of it". ID3

"The visual aspect really works in that case - it does work for me. We can go back to that quote that "one person's death is a tragedy, but that a million is just a statistic".

It's also why I think film and any kind of creative art is, when it is effective, is effective because it takes the personal and makes it universal". ID10

Witnessing pollution damaging wildlife is an emotional nudge to reducing plastic waste

Emotions evoked from seeing wildlife being hurt was a powerful motivator of plastic recycling behaviour. Participants could provide examples of how they had been motivated to engage in recycling behaviour because of their emotional responses to observing how plastic pollution has damaged non-human animal life.

"The animal part touches me, because I'm a vegetarian and I eat a mostly vegan, and it's definitely very sad to see the animals being harmed… I didn't care if it was very hard to see they were showing things that were really gruesome, and that was the turning point for a lot of my friends and I, because you're finally seeing it, it wasn't just plastic on a beach, which is sad, but you're seeing something else suffering because of it, and you feel the impact more, because I mean any kind of living thing you don't want to hurt them, well, most people don't want to hurt them, but definitely the animals, that hits home more especially for me". ID22

"I actually read an article about the typhoon of plastic that is in the oceans at the moment, and how many issues this causes, particularly as well with the highlighted damage of plastic straws, for sea life all these stories together have sort of really highlighted plastic as a negative environmental thing and that is why when I'm thinking about plastic, the first thing I normally think about is all these stories about how animals are being harmed by the production of and wasting of plastic or I should say the incorrect disposal of plastic". ID23

There are limits to what individuals are willing to sacrifice, even among the eco-conscious

In general, participants reported a desire to do the right thing and emphasised the importance of recycling plastics. Indeed, some participants indicated that a clear visual cue such as a label indicating that a product was recyclable would make it more likely that they would purchase that product:

> *"So I have changed products recently to things that specifically market themselves as being completely recyclable, made of recycled products, very low packaging, and they have literally written that, all over it. It was very, very obvious and that completely made me swap my choices". ID8*

> *"I was buying some coffee the other day, beans, coffee beans and it was in a kind of, you know, one of those foil kind of bags that coffee beans come in, and then it had a paper kind of surround which is just a branding thing and I just felt, I just thought, that is just completely… so I didn't buy that, so you kind of start to make some choices about things you cannot buy, and you think actually, that's just pathetic, so you don't buy it". ID12*

However, the effort needed to reduce plastic use was not without limitations, and an important limitation was cost.

> *"If there was a choice, if there was, you can get milk or orange squash or something in a bottle that's biodegradable or one that isn't, then I'd definitely choose the biodegradable one, even if it meant, if it was a little bit more, not if it's like, a lot more, but a little bit more, or something like that ". ID12*

"But now I would probably spend a little bit more, but if it was, the difference of a £1.40 per bottle versus £1 per bottle, I don't think I could get over that, even though it's 40p it's 40% of the price of the alternative". ID23

Community level engagement can endorse recycling behaviours

In addition, some participants also identified the important role of communities in influencing behaviour. Here, the emphasis was on social modelling rather than imposition.

"I have got a lot of friends who are quite ecologically conscious and to a large extent are vegans for ecological reasons or have been lifelong vegetarians and don't own cars and things for ecological reasons… so that's the sort of notion of being on the periphery of this community and knowing about its existence, it's sort of it's nice in general to know that there is a collection of people with shared, well, all collections of people or individuals with shared values that form a group but there's some group formation now, I think more so than ever around ecological and green sustainability issues". ID23

"If community leaders were trying to do something, I think, if these problems were framed as a problem for our community, then, and there might be ways of, sort of massaging the problem such that you talk about impact on the local community. The trouble is of course that it's not on the local community, not at all, there is a wider impact, but there might be a way of appealing to people's civic pride at a local community level and dealing with things that way for instance". ID4.

Acting on impulse

Many of our routine household activities such as shopping and disposing of waste are performed without much thought [41]. Consequently, breaking old habits such as being seduced by attractive containers while shopping instead of trying to avoid plastic packaging, is not easy to stop without some conscious effort.

> *"And also being environmentally friendly too, they see, for example my friend said don't do that because dot, dot, dot and all the information was there, but all the information was there, but I was not conscious, I was not awake but she told me this, this, and this and she showed me how to do it. So then I was ok, this is right, ok, I am going to change that" ID16.*

> *"If strawberries come in, like a very light punnet, or if they're all wrapped up and made to look very nice and presentable, I think I would still go for the very nice and presentable ones, I would give up my ethics - unless I am consciously thinking about it of course" ID1.*

A further limitation was that participants reported acting on impulse when shopping. In other words, not taking the time to determine which products are packaged in recyclable material, but buying products out of habit, or because the packaging makes them convenient, or whatever product is most accessible, given that they are often shopping under time constraints.

> *"While in the supermarket, the different levels of decisions whenever I'm in the supermarket I wouldn't ever think that deeply about it, I don't think I would make decisions primarily on the anticipation of the eating and preparing the food and not acknowledging packaging". ID23*

"In terms of packaging, the kind of things I would buy have quite a short shelf life, and so I wouldn't necessarily pay a lot of attention to packaging…I think in terms of purchasing food, I would say it is about 95% about the actual product, and probably about 5% thinking about the packaging. I think maybe a few years ago, I probably did pay more attention.". ID1

A separate survey was conducted at the same time as this qualitative study and there are some relevant questions from the survey that can help to shed light on the qualitative findings. This survey was online and was designed to examine perceptions about plastic use and plastic food packaging in particular. There were 4 questions in the survey that are relevant to this study, which examined opportunity and motivation. Responses were received from 756 participants and the findings are presented in Table 2. The survey findings reinforce importance of themes extracted relating to the reduction of physical obstacles to increase accessibility and ease of recycling in practice, and the need to motivate recycling in creative ways.

Table 2: Responses to Online Survey Questions (demographics and recycling behaviour)

Age (years)	**Mean**	**SD**
	40.18	15.60
Gender	**Male**	**Female**
	276 (36.5%)	476 (63%)
Have you ever knowingly put plastic recycling into a general waste bin?	**Yes**	**No**
	477 (63.3%)	277 (36.7%)

	Strongly Agree	Agree	Not Sure	Disagree	Strongly Disagree
It is easy to recycle	41 (5.4%)	208 (27.5%)	80 (10.6%)	330 (43.8%)	94 (12.5%)
Recycling is a priority for me	20 (2.7%)	31 (4.2%)	70 (9.4%)	379 (50.8%)	246 (33%)

Discussion

This study sought to examine the facilitators and barriers to plastics recycling by consumers, using the COM-B model as a guide. The findings of the content analysis reveal that almost two thirds of the data fall under the headings of physical capability and physical opportunity, with psychological capability and reflective motivation accounting for remaining third of the data. There is less evidence in the data about the role of automatic motivation or social opportunity. Overall, the main themes point to a knowledge gap; a lack of understanding and confusion about how to recycle plastic waste properly and unclear recycling instructions. This co-exists with a lack of willingness to seek out such instruction that is provided by local authorities. Witnessing plastic pollution harming marine life and places of natural beauty, for some, is an emotional nudge to reducing plastic waste and could activate personal motivation to recycle. But without these visual cues, our respondents lack sufficient motivation to seek the detailed information needed about local recycling policies and educational materials. The recycling of plastic waste is a complex behaviour and poses specific challenges for householders. The reality is many simply do not want to be faced with uncertainty and confusion when trying to perform what, to them, should be made an easy, effortless behaviour.

Participants in the study indicated that the current labelling on plastic packaging adds to that confusion. The confusion expressed by participants has been found in previous research. Most recently, WRAP (2020) [42] found that the most frequently cited barrier to recycling in the UK is ‘uncertainty about what can/can’t be recycled’. Information/knowledge about what can be recycled has also been identified as one of the four main barriers to recycling in a systematic review [43]. In South Africa, lack of knowledge was the second most cited barrier to recycling, behind lack of time [44]. This latter barrier highlights the importance of information being readily accessible (i.e. clear labelling on the plastic packaging itself). Providing information

about what can be recycled elsewhere (not on packaging) is unlikely to be helpful as people are very reluctant to take the time to familiarise themselves with this information. This information needs to be accessible at the point of plastic recycling. People are less likely to be confused about whether a plastic package can be recycled if they immediately recognise recycling advice when they look at the package [45]. Being faced with uncertainty for some does not result in information seeking behaviour, rather it causes frustration, resulting in the plastic product ending up in general waste. This adds weight to the importance of visual, unambiguous cues about the recycling of plastic packaging to remove barriers in the form of frustration.

Not surprisingly, then, one of the solutions offered by participants, to improve recycling of plastics is to have clearer labels on the packaging. Participants reported that existing labels are unhelpful and can only add to confusion. They prefer clear, unambiguous labels and colour coding was also suggested. Labelling about recycling on packaging has previously been found to be a cause of confusion and a determinant of successful recycling [46].

Colour coding of bins was also suggested as a way of helping to identify which types of recycling waste is placed, in which bin. The colour of bins and other aspects of recycling bin design are associated with appropriate separation of waste and acceptability of a recycling scheme [14, 47]. This issue is considered important enough to involve the public in the design of recycling bins in Greece [48]. Participants in the present study additionally indicated that it 7would be preferable for the colour of bins and what should be placed in that bin to be standardised throughout the country. Again, this is an example of how making things easy at the point of recycling, so that waste separation becomes a habitual process, is desirable. On this basis, Burgess et al., (2021) [49] have recommended using one bin for all household

plastics recycling, although they acknowledged that this will impact on other parts of the recycling process.

Although the participants in the present study were positive about recycling, this turns to negativity when recycling is not easy. In other words, there are limits to the sacrifices or efforts that individuals are prepared to make to recycle plastics. Recycling is also less likely to happen as financial or time costs increase. Our data does not allow us to estimate the strength of this relationship or to determine whether the relationship is linear, but the data suggests that the relationship exists, in some form. Our data also points to the need for a further in-depth investigation of the decision-making processes involved around plastic waste disposal.

Our study signals that participants expected government, manufacturers and retailers to do more to manage the amount of plastic in society. This indicates a belief that addressing the reduction and recycling of plastics is not within the locus of control of the individual. An internal locus of control has been shown to be related to environmentally responsible behaviour [50], so these findings might give some cause for concern. However, more recent research suggests that both internal and external control are associated with pro-environmental behaviour [51]. Therefore, perhaps participants are simply expressing a credible view that everyone involved in the circular plastics economy must play a role in improving the situation.

While successful plastic recycling largely depends on public behaviour, retailers can have a powerful impact on consumers purchasing environmentally conscious products [52], and government levies can be effective in reducing plastic waste [53], although government intervention is not by itself a panacea [54].

What this research highlights, are that contextual factors, such as waste disposal infrastructure, and the characteristics of food packaging, are having a significant negative impact on plastic recycling behaviour. Obvious important action, rather than relying upon websites to convey recycling information to householders and consumers by third parties, is to also model the importance of plastics recycling [55]. So, if third parties (manufacturers, government, retailers) take action to make recycling easier, this could have a twofold impact on consumers' behaviour.

Conclusions and Recommendations

The data suggests that the negative impacts of *not* recycling are easy to set aside because there is no ongoing, visual reminder of this impact and responsibility is not equally shared among all those who have a role to place in the value plastics chain. The recycling of plastic waste is a complex behaviour and poses specific challenges for householders who simply do not want to be faced with uncertainty and confusion when trying to perform what, to them, should be made an automatic, effortless behaviour. It seems having to make an extra effort becomes a barrier, and good intentions to recycle will be set aside. The upshot of this is that visible impact of plastics on the environment needs to feature regularly when promoting plastic waste recycling to the public, and the act itself needs to be made as convenient as possible, to maximise the behaviour.

The primary recommendations from this research, which will involve action by government, manufacturers, retailers and consumers are:

- Plastic packaging should have visual, unambiguous cues about whether it can be recycled.

- Bins should be colour coded to assist with the separation of waste and it would be beneficial if these colours were standardised throughout the country and matched with colour coding on packaging.

Acknowledgements

We thank all the participants for donating their time to help with this study. This work was supported by the ESPRC [Grant number EP/S025545/1].

References

1.American Chemistry Council 2021. Available from:

https://www.americanchemistry.com/default.aspx

2.PlasticsEurope. Plastics - the Facts. 2019. Available from:

https://www.plasticseurope.org/application/files/9715/7129/9584/FINAL_web_version_Plastics_the_facts2019_14102019.pdf

3. Ocean Intelligence. Ocean Intelligence. 2021. Available from https://www.oceangeneration.org/Pages/Category/ocean-academy?gclid=EAIaIQobChMItvn1s6b88wIVYmHmCh2BHQSaEAAYASAAEgJSdfD_BwE

4. Merrington A. Recycling of plastics. Applied Plastics Engineering Handbook. 2nd ed. Elsevier; 2017. pp. 167-189.

5. Ajzen I. The theory of planned behavior. Organ. Behav. Hum. Dec. Process. 1991; 50: pp. 179–211.

6. Ajzen I. (2012). The theory of planned behavior. In: Lange PAM, Kruglanski AW, Higgins ET, editors. Handbook of Theories of Social Psychology; 2012. 1: pp. 438–459. London: Sage.

7. Yurieva A, Dahmen M, Paillé P, Boirala O, Guillaumieb L. Pro-environmental behaviors through the lens of the theory of planned behavior: A scoping review. Resources Conservation and Recycling. 2020; 155: pp. 1-12.

8. Berglund C. The assessment of households’ recycling costs: The role of personal motives. Ecological Economics. 2005; 56: pp. 560–569.

9. Hage O, Söderholm P, Berglund C. Norms and economic motivation in household recycling: Empirical evidence from Sweden. Resource Conservation Recycling. 2008; 53: pp. 155–165.

10. Iyer ES, Kashyap RK. Consumer recycling: Role of incentives, information, and social class. J of Consumer Behav. 2007; 6: pp. 32–47.

11. Oates C, McDonald S. Recycling and the domestic division of labour: Is green blue or pink? Sociology. 2006; 40(3): pp. 417-433.

12. Mee N, Clewes PS, Phillips PS, Read AD. Effective implementation of a marketing communications strategy for kerbside recycling. A case study from Rushcliffe (UK). Resources, Conservation and Recycling. 2004; 41: pp. 1–26.

13. Thøgersen J. Monetary Incentives and Recycling: Behavioural and Psychological Reactions to a Performance-Dependent Garbage Fee. J of Consumer Policy. 2003; 26: pp. 197–228.

14. McDonald S, Oates C. Reasons for non-participation in a kerbside recycling scheme. Resources, Conservation and Recycling. 2003; 39: pp.369-385.

15. Barr S. What we buy, what we throw away and how we use our voice. Sustainable household waste management in the UK. Sustainable Development. 2004; 12: pp. 32–44.

16. Carrus G, Passafaro P, Bonnes M. Emotions, habits and rational choices in ecological behaviours: The case of recycling and use of public transportation. J of Environmental Psychology. 2007; 28: pp. 51–62.

17. Do Valle PO, Rebelo E, Reis E, Menezes J. Combining behavioral theories to predict recycling involvement. Environmental Behavior. 2005; 37: pp. 364–396.

18. Onwezen MC, Reinders MJ, Sijtsema SJ. Understanding intentions to purchase bio-based products: The role of subjective ambivalence. J Environ Psychol. [Internet] 2017; 52: pp. 26–36. Available from: http://dx.doi.org/10.1016/j.jenvp.2017.05.001

19. Sijtsema SJ, Onwezen MC, Reinders MJ, Dagevos H, Partanen A, Meeusen M. Consumer perception of bio-based products: An exploratory study in 5 European countries. NJAS - Wageningen J Life Sci [Internet]. 2016; 77: pp. 61–69. Available from: http://dx.doi.org/10.1016/j.njas.2016.03.007

20. Klöckner CA, Matthies E. How habits interfere with norm-directed behaviour: A normative decision-making model for travel mode choice. J of Envir Psychology. 2004; 24: pp. 319-327.

21. Meis J, Kashima Y. Signage as a tool for behavioral change: Direct and indirect routes to understanding the meaning of a sign. PLoS ONE. 2017; 12:8 e0182975.

22. Elster J. Emotions. In: Bearman P, Hedström P, editors. The Oxford handbook of analytical sociology. Oxford University Press New York; 2009; Part 3: pp..51-72.

23. Michie S, Hyder N, Walia A, West R. Development of a taxonomy of behaviour change techniques used in individual behavioural support for smoking cessation. Addictive Behavior. 2011; 36(4): pp. 315-319.

24. Michie S, van Stralen MM, West R. The behaviour change wheel: A new method for characterising and designing behaviour change interventions. Implementation Science. 2011; 6: pp. 6-42.

25. Heidbreder LM, Bablok I, Drews S, Menzel C. Tackling the plastic problem: A review on perceptions, behaviors, and interventions. Sci of Total Envir. 2019; 688: pp. 1077-1093.

26. McNicholas G, & Cotton M. Stakeholder perceptions of marine plastic waste management in the United Kingdom. Ecological Economics. 2019; 163: pp.77-87.

27. Mehta N, Cunningham E, Roy D, Cathcart A, Dempster M, Berry E, Smyth BM. Exploring perceptions of environmental professionals, plastic processors, students and consumers of bio-based plastics: Informing the development of the sector. Sustainable Production and Consumption. 2021; 26: pp. 574-587. https://doi.org/10.1016/j.spc.2020.12.015.

28. Garcia JM, Robertson ML. The future of plastics recycling. Science. 2017; 17(358): pp. 870-872.

29. Ilyas M, Ahmad W, Khan H, Yousaf S, Khan K, Nazir S. Plastic waste as a significant threat to environment – a systematic literature review. Reviews on Envir Health. 2018; 33: pp. 383-406. https://doi.org/10.1515/reveh-2017-0035

30. Burnley S, Coleman T. The environmental and financial benefits of recovering plastics from residual municipal waste before energy recovery. Waste Management. 2018; 79: pp. 79-86. https://doi.org/10.1016/j.wasman.2018.07.034.

31. Tong A, Sainsbury P, Craig J. Consolidated criteria for reporting qualitative research (COREQ): A 32-item checklist for interviews and focus groups. Int J Qual Heal Care. 2007; 19(6): pp. 349–57.

32. Smith JA. Beyond the divide between cognition and discourse: using interpretative phenomenological analysis in health psychology. Psychology & Health. 1996; 11: pp. 261–71.

33. Pistrang N, Barker C. Varieties of qualitative research: A pragmatic approach to selecting methods. In: Cooper H, Camic PM, Long DL, Panter AT, Rindskopf D, Sher KJ, editors, APA handbooks in psychology®. APA handbook of research methods in psychology, 2012. Vol. 2. Research designs: Quantitative, qualitative, neuropsychological, and biological (p. 5–18). American Psychological Association

34. Korstjens I, Moser A. Series: Practical guidance to qualitative research. Part 4: Trustworthiness and publishing. Eur J Gen Pract [Internet]. 2018; 24(1): pp. 120–4. Available from: https://doi.org/10.1080/13814788.2017.1375092

35. Namey E, Guest G, McKenna K, Chen M. Evaluating bang for the buck: A cost-effectiveness comparison between individual interviews and focus groups based on thematic saturation levels. American Journal of Evaluation. 2016; 37(3): pp. 425-40.

36. Hsieh H, Shannon SE. Qualitative Health Research. 2005; 15: pp. 1277-1288.

37. Braun V, Clarke V. Using thematic analysis in psychology. Qualitative Research in Psychology. 2006; 3: pp. 77-101.

38. Gainforth, HL, Sheals K, Atkins L, Jackson R, Michie S. Developing interventions to change recycling behaviors: A case study of applying behavioral science, Applied Environmental Education & Communication. 2016; 15(4): pp. 325-339.

39. Passafaro, Stefano L. Comparing determinants of perceived and actual recycling skills: The role of motivational, behavioral and dispositional factors, Journal of Environmental Education. 2017l; 48(5): pp. 347-356, doi: 10.1080/00958964.2017.1320961

40. Vining J, Ebreo A. What makes a recycler? A comparison of recyclers and non-recyclers. Environmental Behaviour. 1990. 22: pp. 55–73.

41. Verplanken B, Whitemarsh L. Habit and climate change. Current Opinion in Behavioral Sciences 2021; 42: pp. 42–46.

42. WRAP. Recycling Tracker. 2020. Available from https://wrap.org.uk/media-centre/press-releases/recycling-tracker-2020

43. Jesson JK, Stone I. A review of barriers to kerbside recycling household waste in the UK. Aston University. 2009.

44. Strydom WF. Barriers to household waste recycling: Empirical evidence from South Africa. Recycling. 2018; 3(41): pp. 1-23.

45. RECOUP. UK Household Plastics Collection Survey, 2019. Available from https://www.recoup.org/p/89/uk-household-plastic-collection-survey-20192019

46. Buelow S, Lewis H, Sonneveld K. The role of labels in directing consumer packaging waste. Management of Environmental Quality. 2010; 21(2): pp. 198-213. https://doi.org/10.1108/14777831011025544

47. Leeabai N, Areeprasert C, Khaobang C, Viriyapanitchakij N, Bussa B, Dilinazi D, Takahashi F. The effects of color preference and noticeability of trash bins on waste collection performance and waste-sorting behaviors. Waste Management. 2020; 121: pp. 153-163 https://doi.org/10.1016/j.wasman.2020.12.010

48. Keramitsoglou KM, Tsagarakis KP. Public participation in designing the recycling bins to encourage recycling. Sustainability 2018; 10; 1240. https://doi.org/10.3390/su10041240

49. Burgess M, Holmes H, Sharmina M, Shaver MP. The future of UK plastics recycling: One bin to rule them all. Resources, Conservation and Recycling. 2021; 164: No. 105191. https://doi.org/10.1016/j.resconrec.2020.105191.

50. Hines JM, Hungerford HR, Tomera AN. Analysis and Synthesis of Research on Responsible Environmental Behavior: A Meta-Analysis. The Journal of Environmental Education. 1987; 18(2): pp. 1-8.

51. Yang X, Weber A. Who can improve the environment-me or the powerful others? An integrative approach to locus of control and pro-environmental behavior in China. Resources, Conservation and Recycling. 2019; 146: pp. 55- 67.

52.Tsarenko Y, Ferraro C, Sands S, McLeod C. Environmentally conscious consumption: The role of retailers and peers as external influences, Journal of Retailing and Consumer Services 2013; 20(3): pp. 302-310. Available from: https://doi.org/10.1016/j.jretconser.2013.01.006.

53. Martinho, G, Balaia N, Pires A. The Portuguese plastic carrier bag tax: The effects on consumers' behavior. Waste Management. 2017; 61: 3-12. https://doi.org/10.1016/j.wasman.2017.01.023.

54. Walker T, Gramlich D, Dumont-Bergeron A. The case for a plastic tax: A review of its benefits and disadvantages within a circular economy. Wasieleski D.M. Weber J. editors. Sustainability (Business and Society. 360: Vol. 4). Emerald Publishing Limited, 2020 pp. 185-211. https://doi.org/10.1108/S2514-175920200000004010

55. Lin Z, Wang X, Li C, Gordon M, Harder M. Visual prompts or volunteer models: An experiment in recycling. Sustainability. 2016; 8: pp. 458.

Supporting Information file: Appendix A Supplemental Data

Figure

Click here to access/download;Figure;Figure 1.tif

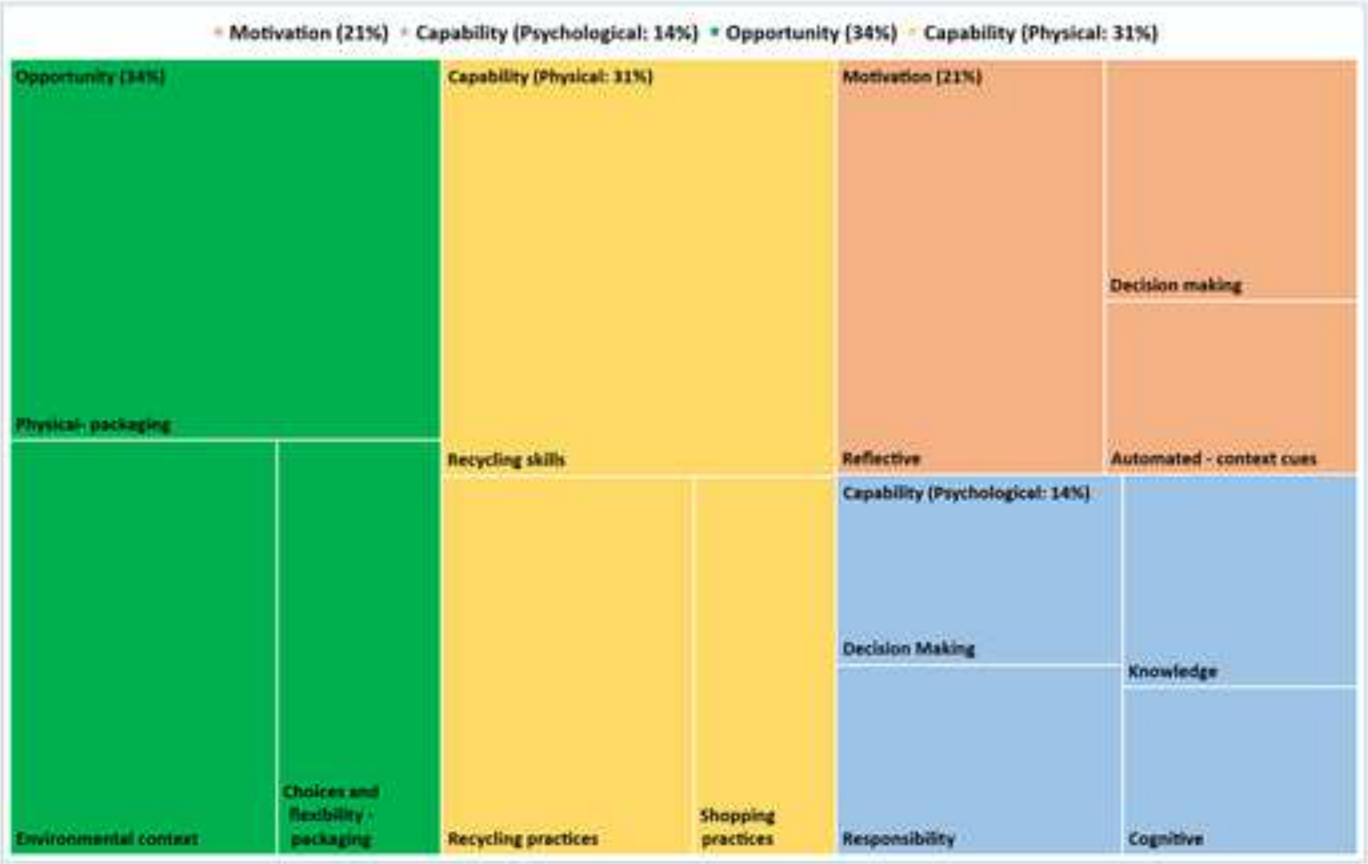

Supplementary data

Click here to access/download
Supporting Information
Appendix A Data Supplemental File 30.3.21.docx
