# Supplementary material for: “If it is not made easy for me, I will just not bother”. A qualitative exploration of the barriers and facilitators to recycling plastics
Source: PLoS One. 2022 May 3;17(5):e0267284. doi: 10.1371/journal.pone.0267284 (PMC9064103; doi:10.1371/journal.pone.0267284)
Supplement: Attachment — Submitted filename: PONE-D-21-36666_comments.docx [file pone.0267284.s004.docx]

Reviewer Comment:

The paper investigates an important issue related to consumer behaviours toward recycling. My comments on the paper are as follow. Additional comments are in the pdf file.

a) Abstract:

An abstract can be reduced by focusing on the aim of the paper, the basic motivation behind it and the key result/message from the paper.

b) Introduction
   a. Shifting the COM-B model to the method section, a clear description of the problem, the gap in the literature and then describing the objective of the study will reduce the confusion and provide clarity on the aim and objective of the study. A thorough revision is necessary.
   b. Through edit of English, especially making it easy to understand by using short sentences.
   c. If the paper is using the COM-B model of behaviours; is it necessary to discuss the TBC model? Why not directly get into the COM-B model. Discussion model not use
   d. A brief literature review or papers showing gaps ( i.e. opportunity factors) would convince the readers on why and how of the study.
c) Method:
   a. The description of the COM-B model should be shifted from introduction to Method by adding a subsection “theoretical framework”
   b. A brief discussion on a) reason to use the model and b) how the model will be used, helps the reader to understand the model and its place in the study.
   c. Also, more clarity on study design would help the reader. A map showing survey covered area would help.
   d. The sample size is too small
   e. Table 2 followed by table 1, recheck the order of the Table.
   f. Table 1 is too simplistic. A table showing detailed characteristics of the respondent (not just sex and age). Also, the author can compare the respondent’s characteristics with national population characteristics if that is helpful for the reader to know about the respondent
   g. Data collection, data analysis and data quality subsection require thorough revision to make it clear and understandable. Also discussing the semi-structured questions in detail, how was it derived and how will be used in the result section has to be clear.
   h. The Ethics subsection is empty
d) Result:
   a. Why and how were the themes selected?
