# Supplementary material for: “If it is not made easy for me, I will just not bother”. A qualitative exploration of the barriers and facilitators to recycling plastics
Source: PLoS One. 2022 May 3;17(5):e0267284. doi: 10.1371/journal.pone.0267284 (PMC9064103; doi:10.1371/journal.pone.0267284)
Supplement: Attachment — Submitted filename: Response to Reviewers..docx [file pone.0267284.s005.docx]

**Response to Reviewers**

Dear Reviewers and Editor for PLOS ONE,

Thank you very much for taking the time to read and comment upon our paper.

Please find a detailed response to each of your comments in the tables that follow, along with a ‘Revised Manuscript with Track Changes’ version of the new manuscript where your suggested changes have been visibly made. Also, we have provided an unmarked final version named ‘Manuscript’. **Please view the ‘All mark-up’ version of the tracked changes document or refer to the 'clean manuscript' in the PDF proof and this way, the line and page numbers will align with each specific comment addressed.**

We have attempted to respond to the comments you made in the PDF version of the submitted manuscript first which is Section 1.

This is followed by our response to the main comments sent to us in a word document. They overlap in some instances, and so we cross referenced where this is the case.

All the changes are in the ‘Revised manuscript with track changes’ paper and so any line and page numbers we provide to direct you relate to this tracked change document and not the final clean manuscript.

Furthermore, we have implemented changes as instructed by the editor: we have adhered to the PLOS ONE file and document formatting; additional information regarding the funders input is as follows “The funders had no role in study design, data collection and analysis, decision to publish, or preparation of the manuscript”; and we have removed funding information from the acknowledgments section in the manuscript.

Dr Emma Berry

Corresponding author

**REVIEW AND RESPONSE TO REVIEWER**

“The paper investigates an important issue related to consumer behaviours toward recycling. My comments on the paper are as follows. Additional comments are in the pdf file”.

1. **SECTION 1 - RESPONSE TO COMMENTS IN PDF DOCUMENT**

| **REVIEWER COMMENTS** | **RESPONSE TO REVIEWER**  **Changes made in Revised Tracked Changed Manuscript** | **Relevant page in PDF** |
| --- | --- | --- |
| **Introduction needs revising as long sentences** | Thank you for your suggestions, – See full response in section 2. | **Page 3** |
| **Inserting reference in mid-sentence** | Page 2, line 29 – reference moved to end of sentence. | **Page 3** |
| **Sentence ending with [3] – long sentence and hard to follow** | Page 2, line 34-36 – sentences simplified. | **Page 3** |
| **3^rd^ sentence from bottom – asks to start new sentence after [5,6]** | New sentence started – see Page 3, Line 56 | **Page 3** |
| **Clearer reference needed to ‘these studies in line 2** | Page 3, line 60-61 is re-written to make the point clearer for readers. | **Page 4** |
| **Is demographics an indirect factor and what is definition of indirect factor** | Please see changes to revised manuscript page 3, lines 60 – 63. | **Page 4** |
| **1^st^ line of 2^nd^ paragraph – no need to cite [5,6] again** | See change in page 3, line 65-67. Citations removed. | **Page 4** |
| **Or, ; ? 6^th^ line up from bottom line** | Not sure what this comment refers to. | **Page 4** |
| **2^nd^ paragraph – hard to follow and understand key point of paragraph** | Paragraph has been re-written to make it easier to read – see page 3 line 93-104 |  |
| **A brief literature review to show gap would be helpful** | See Our response in Section 2 to your point d) concerning the introduction:  additional insights provided from lines 77-100 (page 4). | **Page 4/5 (end of 2^nd^ sentence of2nd paragraph**) |
| **Move 1^st^ paragraph starting with According to Garcia to earlier in paragraph** | Moved. Please see page 2 line 43 | **Page 6** |
| **Move paragraph 3 to methods section** | Moved. Please see Page 6 line 136 | **Page 6** |
| **End of 1^st^ sentence of page “available elsewhere” providing where could help reader** | See Page 6 line 128 and 129 | **Page 7** |
| **Reporting Table 2 first – check ordering** | We have changed ordering of Tables.  see line 165 page 7. | **Page 7** |
| **Revise Data collection paragraph, to make clear and it is not necessary to mention who did what – explain questions that guided semi structured interview** | Thank you. Please see changes made to data collection paragraph in page 7/8, lines 174-188. | **Page 8** |
| **Ethics section is empty** | Complete - see page 8 line 189-192 | **Page 9** |
| **Explain how themes were chosen – 3^rd^ sentence up from bottom of page** | Thank you. We have added more text to explain how Themes were chosen - see page 9 lines 207-216 |  |

**SECTION 2 – RESPONSE TO REVIEWER’S COMMENTS IN MAIN WORD DOCUMENT**

| **REVIEWER COMMENTS** | **RESPONSE TO REVIEWER** |  |
| --- | --- | --- |
| **ABSTRACT** |  |  |
| a)Abstract:  An abstract can be reduced by focusing on the aim of the paper, the basic motivation behind it and the key result/message from the paper. | Thank you for your suggestions. Edits have been made to abstract, to make the purpose and key findings clearer. |  |
| **INTRODUCTION** | **Reply to Reviewer (refer to all marked up version of track changed document)** |  |
| a) Introduction   a: Shifting the COM-B model to the method section, a clear description of the problem, the gap in the literature and then describing the objective of the study will reduce the confusion and provide clarity on the aim and objective of the study. A thorough revision is necessary. | Thank you for your suggestions, the COM-B model description has been shifted to the method section |  |
|  | The gap in the literature is also mentioned in point d) below – see our response |  |
| Introduction  b. Through edit of English, especially making it easy to understand by using short sentences. | Noted  The introduction has been edited significantly and in particular - Page 4 and 5 Line 87-120, has been amended to make it easier to understand. |  |
| Introduction  c. If the paper is using the COM-B model of behaviours; is it necessary to discuss the TBC model? Why not directly get into the COM-B model. Discussion model not use | Thank you. We have taken out a block of text about TPB please to retain a sole focus on the COM-B model.  The discussion section focuses on exploring the themes extracted, which were previously framed around the COM-B model; however we have added the constructs in parentheses when introducing key items of discussion (see page 27 in tracked change manuscript, paragraph 1 in discussion section)  Furthermore, the recommendations provided in the conclusion section suggest intervention functions drawing from the BCW in light of the COM factors identified (see conclusion on page 30 and 31 in discussion section). |  |
| d. A brief literature review or papers showing gaps ( i.e. opportunity factors) would convince the readers on why and how of the study. | Please see additional insights provided from lines 82-118 (page 4,5) which addresses this comment. |  |

| **REVIEWER COMMENTS** | **RESPONSE TO REVIEWER** |  |
| --- | --- | --- |
| **METHOD** |  |  |
| The description of the COM-B model should be shifted from introduction to Method by adding a subsection “theoretical framework” | Moved. Please see Page 5 from line 115 |  |
| A brief discussion on a) reason to use the model and b) how the model will be used, helps the reader to understand the model and its place in the study. | Thank you, a rationale is now provided in page 3 see lines 71- 75 (with reference to methods where the application of the model is further detailed (page 5 from lines 116)). |  |
| Also, more clarity on study design would help the reader. A map showing survey covered area would help. | Not usual practice – those interviewed did not give their addresses, and in some instances travelled some distance to undertake the interview, so it was not possible to provide a map. The survey area is NI wide; inclusive of a broad catchment area. We do however mention that the participants are NI residents. See page 7 lines 158-172 to reflect this. |  |
| The sample size is too small | See page 8, lines 184-189 where justification of adequacy of sample size is provided i.e. “The research team (DR, MD, EB) held coding meetings regularly, and concluded, after 18 interviews, that no new themes of note were emerging and consequently were satisfied that no further information was needed, or saturation point had been reached. Moreover, data saturation was achieved when thematic categories and relationships between categories were comprehensively interrogated and interpreted”. |  |
| Table 2 followed by table 1, recheck the order of the Table. | We have changed ordering of Tables.  see pages 7 and 24. |  |

| **REVIEWER COMMENTS** | **RESPONSE TO REVIEWER** | |
| --- | --- | --- |
|  |  | |
| Table 1 is too simplistic. A table showing detailed characteristics of the respondent (not just sex and the age). Also, the author can compare respondent’s characteristics with national population characteristics if that is helpful for the reader to know about the respondent | Thank you for your suggestions. However, unfortunately we are not aware of a source of information that could give us national population characteristics, nor is it clear what characteristics the reviewer would like us to include. Existing published qualitative research papers around recycling behaviour, to our knowledge, do not offer such comparisons, and so we are not aware of any precedent for reporting this information.  However, what we are able to do, is provide more detail about the occupations and living arrangements of participants.  See page 9, Table 2 | |
|  |  |  |
| Data collection, data analysis and data quality subsection require thorough revision to make it clear and understandable.  Also discussing the semi-structured questions in detail, how was it derived and how will be used in the result section has to be clear. | We have added more text to explain how Themes were chosen - see page 11 lines 256-265 As this analysis is in interpretative in nature – the meanings derived from the coding and categories is subjective, but agreed through consensus, as all researchers independently and then collectively considered the analysis. There was significant agreement as to how they results were to be interpreted.  Please see changes made to data collection paragraph in page 9/10. | |
| The Ethics subsection is empty | Thank you for picking this up.  The Ethics sub section has now been completed. | |
|  |  | |
|  |  | |
| **RESULTS** |  | |
| a.Why, and how were the themes selected? | Thank you for prompting this additional detail. We have added more text to explain how Themes were chosen - see page 11 lines 252-264 | |
