# Supplementary material for: “If it is not made easy for me, I will just not bother”. A qualitative exploration of the barriers and facilitators to recycling plastics
Source: PLoS One. 2022 May 3;17(5):e0267284. doi: 10.1371/journal.pone.0267284 (PMC9064103; doi:10.1371/journal.pone.0267284)
Supplement: Attachment — Submitted filename: 24022022 Plos ONE.docx [file pone.0267284.s006.docx]

The second draft of manuscript is better now, unfortunately I cannot find the specific answers or responses of the authors to my previous comments as copy paste below:

In my opinion, the authors should put the procedure for gathering data in one method first then separate between direct interview and by online. In this case, author mentioned total number of respondents first then separate. In this manuscript, authors seemed set aside online respondents. It was looked from their statement in page 24 (A separate survey...). From this point, authors seemed less satisfaction with number of respondents of direct interview or the results of direct interview. Authors should be neutral, so respondents (both direct interview and online) should have the same position. We have to respect all of their opinion.

Or authors can compare between direct respondents versus online. So in this case statistical analysis are needed.

Further, authors should add a proof or a study that plastics can/ cannot be recycle. Here, terminology of recycle has to be explained whether just re-use, re-pair, or fully re-cycle to be different form, such as become a WPC (wood plastics composite) product. Recycle here means what?

In addition to recycle, did the authors think plastic can be degraded in nature? Issue of microplastics in the environment have been hot nowadays. Some sentences were needed to explain more, such as impact of recycling the plastics (pollution in soil and air/ because of plastics combustion), benefits or drawback of these actions, etc.

Please response these comments first. Thank you.
