# Supplementary material for: “If it is not made easy for me, I will just not bother”. A qualitative exploration of the barriers and facilitators to recycling plastics
Source: PLoS One. 2022 May 3;17(5):e0267284. doi: 10.1371/journal.pone.0267284 (PMC9064103; doi:10.1371/journal.pone.0267284)
Supplement: Attachment — Submitted filename: PONE-D-21-36666-R1- Comments.docx [file pone.0267284.s008.docx]

Comments:

The paper has improved. However, it requires substantive work, especially in the result, discussion and method section. I recommend a major revision. I have marked some comments in the pdf version. Following are the comments by section.

1. Abstract:

The author has revised the abstract. However, the revision is not sufficient. Currently abstract is long. Abstract need further work by directly focusing on the aim of the study, method and the key findings.

2. Introduction:

The introduction has improved but can benefit from one more round of revision especially removing long and complex sentences with the short and meaningful sentences and removing information that doesn’t add the discussion.

3. Method

- The first paragraph, Is this paragraph should be revised to explain the strength of qualitative methods in the COM-B framework? and its advantage for this study using peer-reviewed sources. [This comment is also in the pdf version].
- The author should sufficiently explain Why a semi-structured interview is deemed most appropriate for the study.
- The method section should discuss both the surveys: online and interview and justify why two separate data collection process is implemented and how these two address the research question

4. Result and discussion
   a. Introducing a new online survey in the middle of the result leaves the reader in confusion. Author should discuss all data collection strategies and the reasons for using two data collection strategy in the method section.
   b. Also, the result of the two surveys can be presented in different subsections.
   c. As mentioned, the online survey has 756 respondents. The survey can provide meaningful insight into the study. However, the result is not well presented.
   d. I would suggest the following: Insert a paragraph discussing the survey process (if it is secondary information) mentioning the source of information is helpful. Also, use information in Table 2 like age and sex to discuss the participant characteristics. Then in the result insert an additional subsection and present the result. A chart may be easy to understand.
   e. Also, if there is a separate discussion, the section heading should result only not result and discussion. So doing, the author must shift any discussion from the result section to the discussion section.
   f. Table 2 need revision. Please use a separate table to provide information on a variable with a different structure. For example, responses collected as Likert scale and yes/no(binary) can be separated and presented in a graph chart or different table.
